# Genetic impairment of folate metabolism regulates cortical interneurons and social behavior

**DOI:** 10.3389/fnins.2023.1203262

**Published:** 2023-06-28

**Authors:** Noa Sadigurschi, Gilad Schrift, Johannes Hirrlinger, Hava M. Golan

**Affiliations:** ^1^Department of Physiology and Cell Biology, Faculty of Health Sciences, Ben-Gurion University of the Negev, Beer Sheva, Israel; ^2^Carl-Ludwig-Institute for Physiology, University of Leipzig, Leipzig, Germany; ^3^Department of Neurogenetics, Max-Planck-Institute for Multidisciplinary Sciences, Göttingen, Germany; ^4^Azrieli National Center for Autism and Neurodevelopment Research, Ben-Gurion University of the Negev, Beer Sheva, Israel

**Keywords:** MTHFR, folic acid, interneurons, GABA, neurodevelopment, autism spectrum disorder

## Abstract

**Introduction:**

The implications of folate deficiency in neuropsychiatric disorders were demonstrated in numerous studies. Genetic deficiency in a key folate metabolism enzyme, MTHFR, is an example of the interaction between genetic and environmental risk factors: the maternal MTHFR deficiency governs *in-utero* nutrient availability, and the embryo’s *Mthfr* genotype influences its ability to metabolize folates. Here, we explore how the maternal and offspring *Mthfr* genotypes affect cortical interneuron densities and distributions, mouse social outcome, and the relation of the different interneuron patterns to cortical excitability.

**Methods:**

Two experiments were conducted to examine the effects of maternal and offspring *Mthfr*-KO heterozygosity. Mice were tested for direct social interactions (DSIs), repetitive behavior and cortical laminar distribution of interneuron populations expressing glutamate-decarboxylase-65, parvalbumin and somatostatin. Susceptibility to seizure was tested by exposure to pentylenetetrazole (PTZ).

**Results:**

Maternal *Mthfr+/−* genotype was associated with suppressed social activities and reduced interneuron densities in all layers of the retrosplenial cortex (RSC). Somatostatin density and the somatostatin/parvalbumin ratio in the RSC and frontal cortex positively correlated with social behavior in the mice. An interaction between maternal and offspring *Mthfr* genotypes resulted in higher susceptibility of wild-type offspring to PTZ induced seizure.

**Discussion:**

Maternal folate metabolism was shown to be critical to interneuron ontogenesis. Our results demonstrate that interneurons have a specific susceptibility to folate deficiency that may mediate folate’s involvement in neuropsychiatric disease. The relations between cortical somatostatin interneuron patterns and social behavior highlight this subpopulation of interneurons as a target for further research.

## Introduction

The importance of folate metabolism for brain development and the implications of folate deficiency in neuropsychiatric disorders have been repeatedly demonstrated in a wide range of studies (Lewis et al., 2005; Muntjewerff et al., 2006; Roza et al., 2010; Levine et al., 2018; Zou et al., 2021). In the light of accumulating evidence supporting the beneficial effects of folic acid (FA) supplementation during pregnancy to prevent neural tube defects (MRC Vitamin Study Research Group, 1991; Wilson and O’Connor, 2021), FA fortification was recommended in 1992 by the CDC for woman of childbearing ages. Epidemiological studies have further supported FA’s potential to reduce the risks of giving birth to a baby that will be diagnosed later in life with autism spectrum disorder (ASD) (Levine et al., 2018). Moreover, an interaction between maternal FA intake and maternal polymorphism in a gene coding for key folate metabolism enzyme, methylene-tetrahydrofolate reductase (*Mthfr, Mthfr677C > T*, rs1801133), was reported to rescue the risk for neurodevelopmental outcome in the newborn (Schmidt et al., 2011, 2012; Pu et al., 2013; Rai, 2016).

Considering that maternal *Mthfr* genetics and FA intake contribute to the psychiatric outcome of the newborn *via* a single metabolic pathway, the *Mthfr677C > T* polymorphism constitutes an example of gene–environment interaction. In this case, the maternal polymorphism is an environmental risk factor that leads to an MTHFR-deficient intrauterine environment, and the presence of the polymorphism in offspring is a genetic risk factor. The maternal MTHFR deficiency not only reduces the availability of various metabolites in the intrauterine environment, it also dictates placental gene expression (Luan et al., 2021). The significance of the FA metabolic pathway to brain development is further supported by the interactions with other genetic variations (*Mthfr1289A > C*, rs1801131), FA dosage and the presence of folate receptor autoantibodies (Hoxha et al., 2021).

The folate cycle coupled with the methionine cycle is referred to as one carbon metabolism. Indications that one carbon metabolism function was disturbed due to the *Mthfr677C > T* polymorphism in humans or the *Mthfr* haploinsufficiency (*Mthfr+/−* genotype) in mice were previously shown (Chew et al., 2011; Yan et al., 2011). Moreover, the effects of maternal and offspring *Mthfr+/−* genotype on methionine, betaine and choline levels in the cerebral cortex and basal ganglia were associated with mice behavior (Orenbuch et al., 2019).

Dysregulation of the excitation-inhibition balance in brain circuits was suggested to be an underlying mechanism for autism and schizophrenia (Marenco and Weinberger, 2000; Cotter et al., 2002; Lewis and Hashimoto, 2007; Charych et al., 2009; Taylor and Tso, 2015; Buckley and Holmes, 2016; Frye et al., 2016; Jacob, 2016; Kumar et al., 2021). A weak inhibitory tone in ASD is supported by vast evidence of the differences that have been observed in the inhibitory cells, proteins and gene expression patterns in the postmortem brains of ASD patients (Fatemi et al., 2002, 2014; Oblak et al., 2010; Gaetz et al., 2014; Hashemi et al., 2016; Robertson et al., 2016; Hong et al., 2020; Satterstrom et al., 2020; Amina et al., 2021) and in animal models of ASD (Eagleson et al., 2010; Jedlicka et al., 2011; Han et al., 2012; Wohr et al., 2015; Filice et al., 2016). Moreover, it appears that several of the genes associated with ASD, such as Scn1A, Shank1 and Dlx1/2, are expressed preferentially in inhibitory interneurons during an early developmental window (Cobos et al., 2005; Han et al., 2012; Mao et al., 2015). The largest classes of cortical interneurons are characterized by the expression of parvalbumin (PV) and somatostatin (SST), which differ in their laminar and targets of connectivity (Pfeffer et al., 2013; Tremblay et al., 2016). PV and SST interneurons play important roles in the initiation and maintenance of cortical beta-gamma band oscillations in the resting state and upon sensory activation (Kato et al., 2015; Hayden et al., 2021), wherein they exhibit a layer specific response (Kuki et al., 2015). Proper interneuron incorporation in the cortical circuits is a fundamental prerequisite for orchestrated function.

Our previous studies linked the mouse *Mthfr+/−* genotype to ASD-like behavior and low levels of GABA pathway proteins in the cerebral cortex (Sadigurschi and Golan, 2018; Orenbuch et al., 2019). Here, we broaden the examination of the maternal and offspring *Mthfr* genotypes, and the association between ASD-like behavior and the density and laminar distribution of cortical interneurons.

## Materials and methods

### Mouse colonies

Two mouse colonies were raised: *heterozygous Mthfr-KO mice* (*Mthfr+/−*) on a Balb/cAnNCrlBR background (Chen et al., 2001), and *GAD65-tdTomato: Transgene GAD65-tdTomato mice* on a C57/Bl6 BAC background (Besser et al., 2015). The colonies were maintained on a 12:12 h light/dark schedule, and food and water were provided *ad libitum*. All procedures were performed according to the guidelines of the Israeli Council on Animal Care and approved by the Animal Care and Use Committee of Ben-Gurion University of the Negev (protocols IL-16-07-14 and IL 10–03-18D).

### Study design

#### Experiment 1

To assess the effects of the maternal *Mthfr+/−* genotype vs. the offspring genotype, the tested mice comprised the first generation (F1) produced by *Mthfr+/−* (HT) and *Mthfr+/+* (WT) females with GAD65-tdTomato males (WT). Three groups are represented by the genotype of maternal:paternal:offspring: (group 1) WT offspring of WT parents (WT:WT:WT, *N* = 6), (group 2) WT offspring from pairs of HT dams and WT males (HT:WT:WT, *N* = 8), and (group 3) HT offspring produced by HT dams and WT males (HT:WT:HT, *N* = 8).

#### Experiment 2

To exclude the *in-utero* contribution of the *Mthfr+/−* genotype to the phenotype, we mated GAD65-tdTomato (WT) females with *Mthfr*+/− males to create two additional groups – (group 4) WT offspring of WT dams and HT males (WT:HT:WT, *N* = 10), and (group 5) HT offspring of WT dams and HT males (WT:HT:HT, *N* = 10). During pup lactation, cages were observed and adverse or neglect maternal care was excluded. Tests were performed on 3-month-old mice, not more than 2 offspring from each litter/group were included.

**Figure 1:**
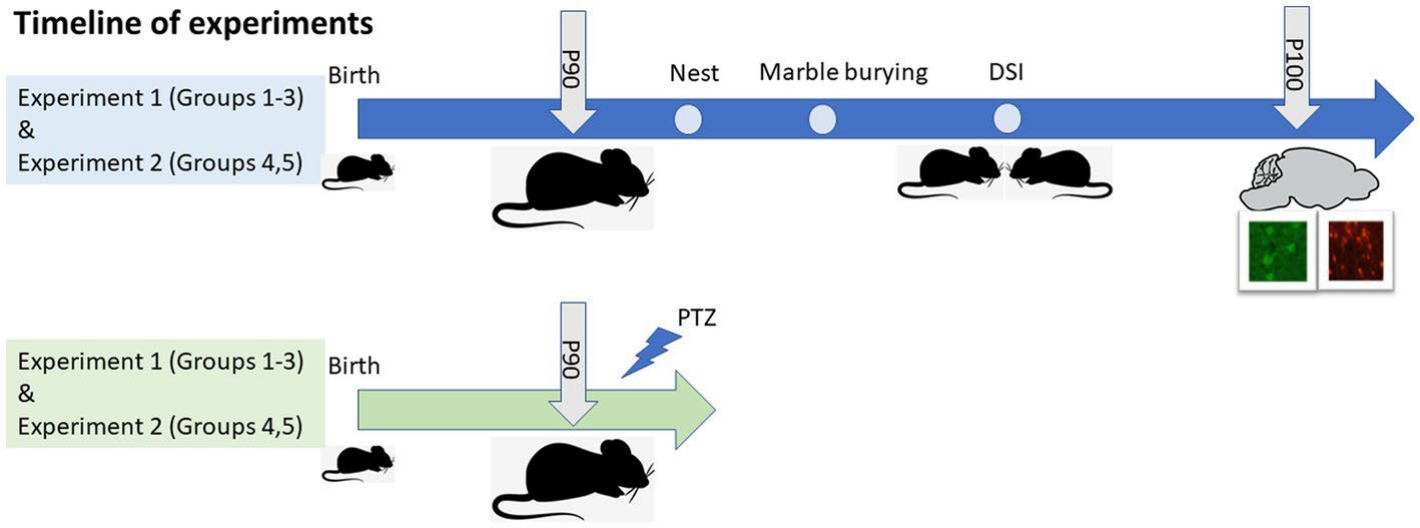
Experimental design and timeline of experiments. Two batches of mice were used for the study, presented in blue and green. P, Postnatal day; DSI, Direct social interaction; PTZ, pentylenetetrazol.

We generated an additional batch of similar groups for the seizure susceptibility test. Mice were tested beginning on postnatal day 90, as shown in Figure 1. We previously showed that the maternal and offspring *Mthfr+/−* genotypes were associated with an ASD-like phenotype in male mice, and minor effect in female mice (Sadigurschi and Golan, 2018). In the current study, therefore, we focused on male mice.

### Genotyping

Mothers and offspring were genotyped as described previously by using polymerase chain reaction amplification of DNA isolated from toe clips. The primers used were as follows: *Mthfr*: sense primer 1 (5′-GAAGCAGAGGGAAGGAGGCTTCAG-3′) in exon 3, sense primer 2 (5′-AGCCTGAAGAACGAGATCAGCAG C-3′) in the neo^r^ gene, and antisense primer 3 (5′-GACTA GCTGGCTATCCTCTCATCC-3′) in intron 3. *GAD65-tdTomato*: Sense primer 1 (5′-GTGCAGGGTCGAGGCAAAGGCA-3′), and antisense primer 2 (5′-GGACAGGATGTCCCAGGCGAAG-3′) (Chen et al., 2001; Besser et al., 2015).

### Behavioral assessments

#### Nest building

Animal welfare and repetitive behavior were assessed based on several nest features (Gaskill et al., 2013; Sadigurschi and Golan, 2018). Prior to testing, mice were separated and placed in individual cages. Nest material comprised tissue paper folded into a fixed size of 5 × 7 cm and placed in each cage. The size and quality of the nests were measured 24 h after the insertion of the bedding. Scores (0–3) were given for material processing, centralization and symmetry. The volume of the nest was calculated by length × width × height.

#### Marble burying test

Repetitive behavior was additionally evaluated by recording the number of buried (“hidden”) marbles (Thomas et al., 2009). Each mouse was placed in a cage with 15 marbles organized in 5 × 3 rows for a duration of 10 min. At the end of the test the mouse was removed from the cage. Each cage was photographed before mouse introduction into it and after mouse removal from it. The number of buried marbles was counted.

#### Direct social interaction test

To measure sociability, two mice from the same group were inserted into a clean cage where their behavior was then videotaped for 15 min. The following behaviors were measured offline (duration and number of occurrences) by a blinded experimenter as previously described (Defensor et al., 2011; Sadigurschi and Golan, 2018) with some modification to the types of interactions that were tested:

Sniffing: Different aspects of sniffing of the companion mouse were quantified:

Nose tip-to-nose tip: Subject’s nose tip and/or vibrissae contacts the nose tip and/or vibrissae of the other mouse.

Nose-to-head: Subject’s nose or vibrissae contacts the dorsal, lateral, or ventral surface of other mouse’s head.

Nose-to-anogenital: Subject’s nose or vibrissae contacts the base of the tail or anus of the other mouse.

Body Sniffing: Subject’s nose tip and/or vibrissae contacts body areas not specified before.

In addition, play and grooming were quantified by the following:

Whisker trimming: Representative of grooming and dominant behavior.

Crawl Over: Subject’s forelimbs cross the midline of the dorsal surface of the other mouse.

Crawl Under: Subject’s head goes under the ventral surface of the other mouse to a depth at which at least the ears of the subject animal cross the midline of the other mouse’s body.

Because the crawl over and crawl under behaviors are fast events, only the number of events was analyzed.

All sniffing and play aspects were considered Non-Aggressive behaviors.

Aggressive behaviors were quantified by:

Attack: Initiation of aggressive behavior.

Fight: Participation in an aggressive event during which the two mice tussle violently and the observer is unable to distinguish the subject mouse from the other mouse.

Aggressive behavior: Sum of attacks and fighting events.

#### Behavioral phenotype

Mice were phenotyped based on how social and repetitive behaviors manifested themselves in the animals.

We used the Direct social interaction (DSI) test to categorize mouse behavior as either (a) “Social” or (b) “Non-social” in mice that engaged in sniffing behavior for longer or shorter times, respectively, than the median duration for sniffing behavior.

We evaluated repetitive behavior with the marble burying test. Mouse behavior was classified as either (a) “Non repetitive” or (b) “Repetitive” in mice that hid fewer or more marbles, respectively, than the median number of marbles hidden.

The categories thus determined for mouse social and repetitive behaviors were used to define “ASD-like” behavior (i.e., mice that presented sniffing times of longer durations than the median and that hid larger numbers of marbles than the median) and “non-ASD-like” behavior (i.e., mice that presented sniffing times of shorter durations than the median and that hid smaller numbers of marbles than the median). In addition, we defined a third group of “partially symptomatic” mice (i.e., sniffing times of shorter durations than the median or numbers of marbles hidden larger than the median).

#### Immuno-fluorescence analysis of brain tissue

Mice interaction with the experimenter and the participation in the DSI test may induce a stress response, which could include gene regulation, glia activation and more. To avoid the effects of these factors on the variables measured in brain tissue, after the completion of the DSI test, we allowed the mice to spend a week in their home cage basal conditions before sampling brain tissue. Mice were anesthetized by inhalation of 30% isoflurane (Minrad Inc., NY, United States) diluted in isopropanol (Fluka Chemie GmbH, Buchs, Netherlands), after which they were transcardially perfused with paraformaldehyde (PFA) 4%. Brains were rapidly removed into a 4% PFA solution and stored at 4°C overnight. Each brain was then washed in phosphate buffer and transferred to a 10% sucrose solution for 2 h or until it sank to the bottom of the solution. Afterwards, brains were transferred to a 30% sucrose solution for 24 h at 4°C and then immersed in OCT compound embedding matrix (Tissue-Tek #4583) inside cryo standard plastic vinyl disposable molds (25 × 20 × 5 mm) placed on dry ice and stored at −80°C. The brains were sliced into 10-μm thick sagittal sections between the bregma lateral plane, 0.24–0.36 mm. Neurons were examined by using the following primary antibodies: Polyclonal guinea pig anti-NeuN (Millipore, 1:400), Monoclonal mouse anti-PV (1:3,000, Sigma-Aldrich, Cat # P 3088), Monoclonal mouse anti-SST (Santa Cruz Biotechnology, 1:250), and Polyclonal rabbit anti-RFP (1:500, Rockland, Cat# 600–401-379) was used to detect the tdTomato expression that occurred under the regulation of the GAD65 promoter, and therefore, it marked all interneurons. In addition, Monoclonal mouse anti-NKCC1 [(T4), 1:500, DSHB] and Monoclonal mouse anti-Gephyrin (Synaptic system, 1:1,000). The following were used as secondary antibodies: Goat anti-rabbit Cy3 (1,600, Chemicon, Cat # AP-124C), Donkey anti-mouse Alexa488 and Donkey anti-guinea pig Alexa488 (Jackson Immuno Research, 1:400). Images were taken using a PD73 Olympus CCD attached to an IX-70 Olympus fluorescence microscope and then collected by CellSense software (Olympus) at a × 20 magnification. Images of the frontal cortex (FC), retrosplenial cortex (RSC) and hippocampus regions were captured from each brain section and examined (see Supplementary Figure S1). The analysis was performed using NIH image J. software by an experimenter blind to the tissue identity. For the analysis, cortical images from the pia to the ventricle were combined and divided into 10 bins of equal sizes for cell density and fluorescence optical density analyses. In each bin, the number of cells and the fluorescence optical density were analyzed to calculate cell density and innervation, respectively. Cortical layers (L) were defined as follows: L1 (bin 1), L2 (bins 2–3), L3 (bins 4–6), L4 (bin 7) L5 (bin 8), L6 (bins 9–10). Hippocampal images were divided by fields and cellular region as follows: CA1 and CA3 were divided into the stratum oriens (SO), stratum pyramidale (SP) and stratum radiatum (SR), and the dentate gyrus (DG) was divided into the hillus and stratum granulare (SC) and stratum moleculare (SM).

#### Seizure induction by PTZ

Mice were transferred into new individual cages and injected with 50 mg/Kg of the GABA blocker pentylenetetrazole (PTZ, Sigma-Aldrich, CAS 54–95-5) diluted in saline. Mice were observed for the following 20 min, and the seizure intensities were recorded according to a five-score scale described by Powell et al. (2003).

- 1- Ear and facial twitching.
- 2- One or more myoclonic twitches of the whole body.
- 3- A weak to moderate generalized clonic seizure without loss of righting reflexes.
- 4- Generalized clonic convulsions with rearing and falling down episodes.
- 5- Clonic seizures with loss of righting reflexes followed by tonic hindlimb extension.
- The highest score during each minute was recorded.

### Statistical analysis

Statistical analysis was performed using SPSS 26 software. Univariate two-way ANOVA analysis was used to test the effects of the independent factors, genotype and maternal genotype, and ANOVA for repeated measurement was used when applicable. A two-tailed Student’s *t*-test was used to analyze the effect of genotype in experiment 2. Correlation between cell density and behavior was tested by Spearman’s correlation test. Differences with *p*-values <0.05 were regarded as significant. Data are presented as means ± SEM.

## Results

ASD-like behavior in adult and pup mice was associated with maternal and offspring *Mthfr+/−* genotypes (Sadigurschi and Golan, 2018; Orenbuch et al., 2019; Agam et al., 2020; Shekel et al., 2021). Since previous studies have been done with the balb/c strain, which is known for low sociability, here we crossed these mice with the C57/Bl6 strain, which is known for its sociability, to generate the experimental groups. In addition, to isolate the effect of offspring genotype, WT and HT offspring of WT dams were tested (experiment 2).

### Maternal *Mthfr+/−* genotype is associated with enhanced ASD-like behavior in the offspring

**Figure 2:**
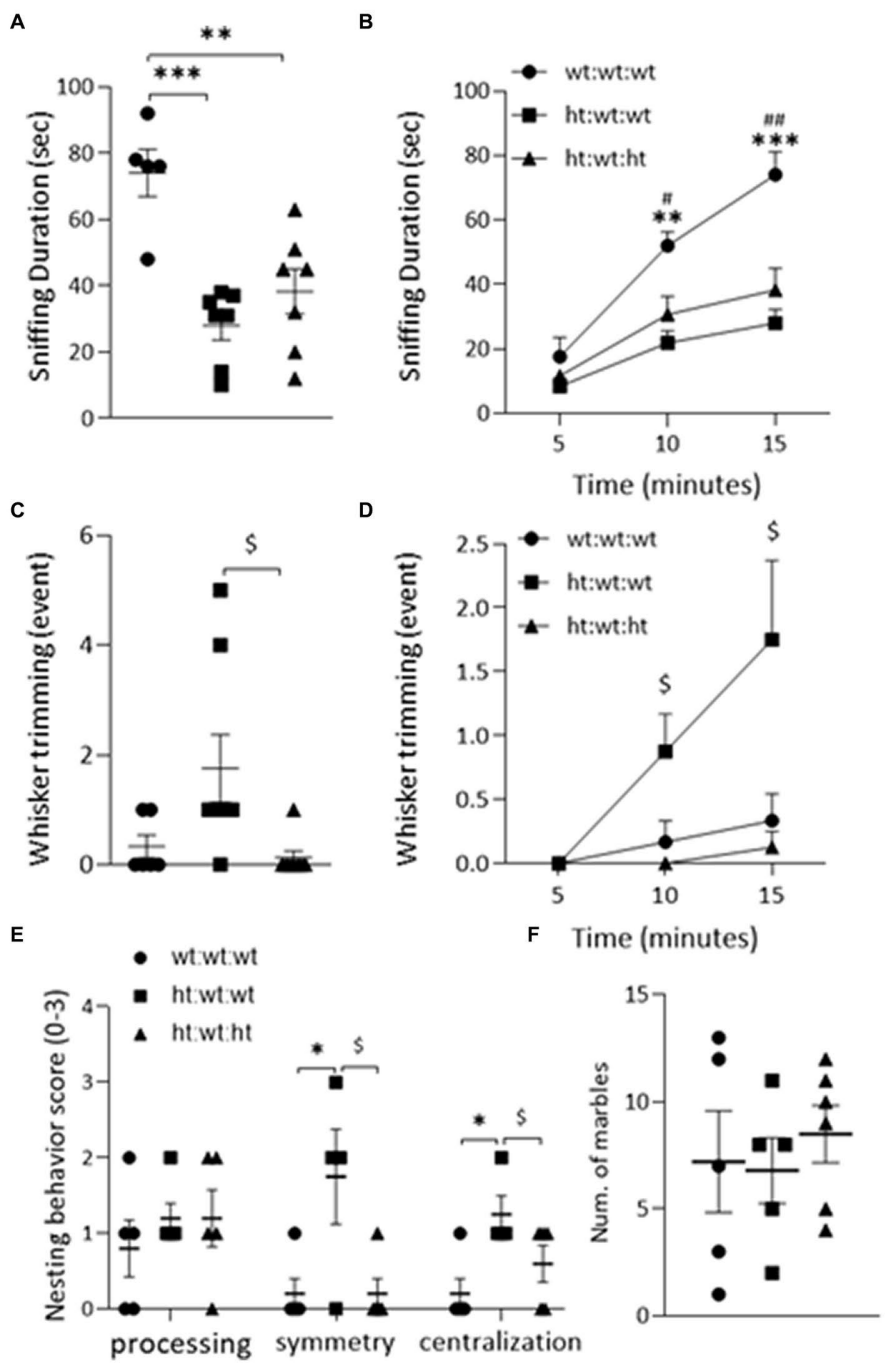
ASD-like behaviors observed in adult mice. *Sociability* was evaluated by the “DSI” test **(A,B)**. The duration of “Sniffing” behavior was decreased by maternal *Mthfr+/−* genotype in the total 15 min of the test (*F*_1,18_ = 26.54, *p* < 0.001, two-way ANOVA) **(A)** and when analyzing the cumulative time (*F*_1,16_ = 20.15, *p* = 0.005, ANOVA for repeated measures) **(B)**. **(C)** The number of “Whisker-trimming” events was increased by maternal *Mthfr+/−* genotype and decreased by offspring *Mthfr+/−* genotype (*F*_1,21_ = 5.5, *p* = 0.03, *F*_1,21_ = 8.46, *p* = 0.009, respectively, two-way ANOVA), and when analyzing the cumulative number of events (*F*_1,19_ = 6.09, *p* = 0.023, *F*_1,19_ = 20.15, *p* < 0.001, respectively, GLM for repeated measures) **(D)**. *Repetitive behavior* was assessed by the “Nest building” **(E)** and the “Marble burying” tests **(F)**. Nest symmetry was increased by maternal *Mthfr+/−* genotype and decreased by offspring *Mthfr+/−* genotype (*F*_1,13_ = 9.24, *p* = 0.011, *F*_1,13_ = 9.24, *p* = 0.01, respectively, two-way ANOVA). Nest centralization was increased by maternal genotype (*F*_1,13_ = 9.8, *p* = 0.01). Data are presented as means ± SEM. For the Sociability test, the N for groups 1, 2, & 3 = 6, 8, & 8, respectively. For the repetitive behavior tests, *N* = 6 per group. A one-way ANOVA with a Bonferroni *post-hoc* test *p < 0.05, **p < 0.01, ***p < 0.005 between wt:wt:wt and ht.:wt:wt, #*p* < 0.05, ##*p* < 0.01 between wt:wt:wt and ht.:wt:ht., $*p* < 0.05 between ht.:wt:wt and ht.:wt:ht. When averaged data are shown, bars represent SEM.

Sociability: The total duration of sniffing was suppressed by the maternal *Mthfr+/−* genotype from 62.6 ± 31.2 s in the wt:wt:wt group to 32.6 ± 16.7 s and 42.8 ± 21.1 s in the offspring of *Mthfr+/−* dams (ht:wt:wt and ht.:wt:ht., respectively, *F*_1,18_ = 26.5, *p* < 0.001) as shown in Figures 2A,B and Table 1. The duration of all non-aggressive behaviors in the wt:wt:wt groups, 81 ± 21.8 s, was longer than those of *Mthfr+/−* dam offspring in the ht.:wt:wt and ht.:wt:ht. groups, 35.3 ± 16.5 s and 55.6 ± 32.5 s, respectively (*F*_1,20_ = 10.2, *p* = 0.005). The similar number of non-aggressive events observed in the latter two groups led us to conclude that the duration of each non-aggressive event was shorter in the affected mice. Whisker trimming, representing a dominant behavior in the mice was differentially affected by the maternal and offspring genotypes, such that it was elevated by the maternal and suppressed by the offspring *Mthfr+/−* genotype (*F*_1,21_ = 5.5, *p* = 0.03, *F*_1,21_ = 8.46, *p* = 009, respectively Figures 2C,D).

**Table 1:** Direct social behavior between two mice of the same group.

	Experiment 1—groups averages			Statistics			Experiment 2—groups averages		
Behavior type	wt:wt:wt	ht:wt:wt	ht:wt:ht	Univariate analysis	Anova	*Post hoc*	wt:ht:wt	wt:ht.:ht	*T-*tests
N	6	8	8				10	10	
NN (s)	4.2 ± 2.588	1.857 ± 1.864	0.816 ± 0.308	MG:*F*_1,21_ = 3.73, *p* = 0.068	NS		2 ± 0.707	1.125 ± 1.125	NS
				G: NS					
NH (s)	6.6 ± 6.767	4.285 ± 6.701	1.142 ± 1.214	MG: NS	NS		4.111 ± 4.781	2.444 ± 3.469	NS
				G: NS					
NA (s)	13.2 ± 13.989	6.142 ± 7.670	15.875 ± 8.559	MG:*F*_1,21_ = 3.43, *p* = 0.08	NS		9.333 ± 6.670	4.777 ± 4.576	NS
				G: NS					
BS (s)	30 ± 27.151	16.571 ± 10.097	17.571 ± 10.485	G: NS	NS		14.777 ± 9.640	13.111 ± 12.128	NS
				MG: NS					
Total sniffing (s)	62.666 ± 31.232	32.625 ± 16.758	42.875 ± 21.076	G:NS	p < 0.001	1–2: p < 0.001	45.6 ± 133.649	29.8 ± 28.751	NS
				MG:*F*_1,18_ = 26.54, *p* < 0.001		1–3: *p* = 0.003			
CU (events)	0.4 ± 0.894	0.2854 ± 0.487	0.428 ± 0.786	G: NS	NS		0.777 ± 1.092	0.777 ± 1.092	NS
				MG: NS					
CO (events)	0.6 ± 0.894	0.5 ± 0.534	0.75 ± 0.886	G: NS	NS		0.666 ± 0.5	0.444 ± 0.726	NS
				MG: NS					
WT (events)	0 ± 0	0.571 ± 0.786	0 ± 0	G: *F*_1,21_ = 8.46, *p* = 0.009	*p* = 0.02	1–2: *p* = 0.09	0.111 ± 0.333	0 ± 0	NS
				MG: *F*_1,21_ = 5.5, *p* = 0.03		1–3: *p* = 0.027			
Non-aggressive behavior (s)	81 ± 21.874	35.5 ± 16.518	55.625 ± 32.522	G: NS	*p* = 0.017	1–2: *p* = 0.015	48.2 ± 34.637	26 ± 21.621	NS
				MG: *F*_1,20_ = 10.21, *p* = 0.005					
Non-aggressive behavior (events)	26 ± 7.314	19 ± 7.23	23 ± 8.847	G: NS	NS		24.7 ± 11.136	18 ± 13.802	NS
				MG: NS					
Attack (s)	0.167 ± 0.408	3.125 ± 5.792	1.625 ± 4.596	G: NS	NS		1.2 ± 3.794	6.2 ± 8.753	NS
				MG: NS					
Fight (s)	2.666 ± 4.131	0.25 ± 0.707	0 ± 0	G: NS	NS		0 ± 0	0.5 ± 1.269	NS
				MG: *F*_1,21_ = 4.28, *p* = 0.052					
Aggressive behavior (s)	2.833 ± 4.4	3.375 ± 6.254	1.625 ± 4.596	G: NS	NS		1.2 ± 3.794	6.7 ± 6.718	NS
				MG: NS					

Mice behavior during 15 min of the direct social interaction was quantify by the following behaviors: NN, Nose tip-to-nose tip; NH, Nose-to-head; NA, Nose-to-anogenital; BS, Body sniffing; CO, Crawl Over; CU, Crawl Under; WT, Whisker trimming; attack and fight. Sniffing = sum of NN + NH + NA + BS. Non-Aggressive behavior = Sniffing+CO + CU + WT. Aggressive behavior = Attack + fight. OG, offspring *Mthfr* genotype; MG, maternal *Mthfr* genotype; NS, not significant. For *post hoc* analysis, the experimental groups wt:wt:wt, ht.:wt:wt and ht.:wt:ht. were referred as 1, 2, 3, respectively. Data are presented as means ± SD.

Processing of nesting material as representative of repetitive/rigid behavior highlighted the effect of maternal genotype. Nest symmetry was increased by the maternal *Mthfr+/−* genotype and decreased by the offspring *Mthfr+/−* genotype (*F*_1,13_ = 9.24, *p* = 0.011, *F*_1,13_ = 9.25, *p* = 0.01, respectively), and nest centralization was increased by maternal genotype (*F*_1,13_ = 9.8, *p* = 0.01, Figure 2E). Similar performances were observed in all of the groups in the marble burying test (Figure 2F).

Similar behavioral analyses of the *Mthfr+/+* and *Mthfr+/−* offspring of *Mthfr+/+* dams did not show any effect of offspring genotype on adult mouse behavior (see Supplementary Figure S2 and Table 1), a finding that emphasizes the significance of the *in-utero* environment.

Taken together, the findings show that the maternal *Mthfr+/−* genotype had significant effects on social and repetitive behaviors in the F1 offspring.

### Maternal *Mthfr+/−* genotype is associated with cortical interneuron defect

**Figure 3:**
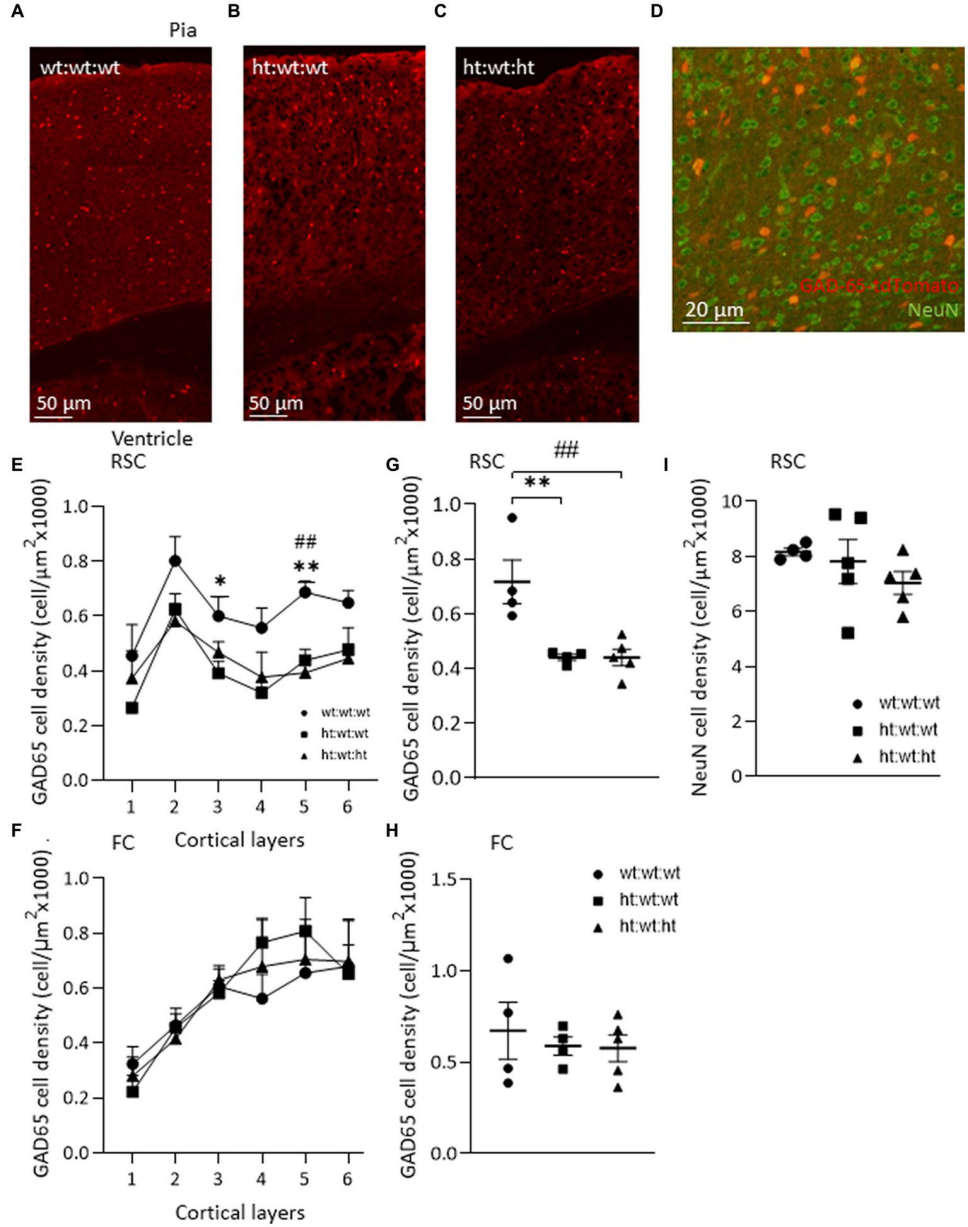
Maternal *Mthfr*+/− genotype is associated with interneuron deficiency in mice RSC. **(A–C)** Images of/ GAD65-tdTomato interneurons in the RSC of each experimental group, from top – pia, to bottom – ventricle. **(D)** An image of GAD65-tdTomato (red) and NeuN^+^ neurons (green) in the RSC. **(E,G)** *GAD65-tdTomato interneuron density* was reduced by the maternal *Mthfr+/−* genotype in the RSC (*F*_1,12_ = 15.84, *p* = 0.003, two-way ANOVA). The decrease in cell density was observed in all layers, most prominently in layers 3 and 5 (*F*_1,10_ = 36. 4, *p* < 0.001, ANOVA for repeated measures, *F*_1,12_ = 7.83, *p* = 0.019, *F*_1,12_ = 16.74, *p* = 0.002 two-way ANOVA, respectively). **(F,H)** A similar analysis of GAD65-tdTomato in the FC revealed no difference between groups. **(I)** *NeuN cell density* in the RSC. Data are presented as means ± SEM, N of group 1 = 4, of group 2 = 5, of group 3 = 5. One-way ANOVA with a Bonferroni *post-hoc* test *p < 0.05, **p < 0.01 between wt:wt:wt and ht.:wt:wt, ##*p* < 0.01 between wt:wt:wt and ht.:wt:ht.

To explore the possible association of the above-described behaviors with interneuron dysregulation, the brains of the mice tested above were analyzed. The expression of GAD65-tdTomato in interneurons in the RSC region, and colocalization of GAD65 and NeuN staining are shown in Figures 3A–D. Comparisons of the laminar distributions of GAD65-tdTomato interneurons sampled from the RSC and FC regions showed that their respective distribution patterns differed in these brain regions (Figures 3E,F). GAD65-tdTomato interneuron density across the entire RSC, was suppressed by the maternal *Mthfr+/−* genotype (*F*_1,12_ = 15.84, *p* = 0.003), and the effect was proportional in all cortical layers (*F*_1,10_ = 36.4, *p* < 0.001, ANOVA for repeated measures) (Figures 3E,G). The maternal *Mthfr+/−* genotype had strong effects in layers 3 and 5 (*F*_1,12_ = 7.83, *p* = 0.019, *F*_1,12_ = 16.74, *p* = 0.002, respectively). In the FC, GAD65-tdTomato interneuron densities in layers 4 and 5 were non-significantly higher in the offspring of *Mthfr+/−* dams compared to that in the wt:wt:wt group, but all groups exhibited similar total densities (Figures 3F,H). Lastly, the effects of the maternal and offspring genotypes on interneuron density in the hippocampus, restricted to the CA1 and DG regions, are shown in Table 2 and Supplementary Figure S3. In contrast to the interneurons, NeuN+ cells were found in similar densities in both the RSC and FC regions and in all the groups (Figure 3I). Thus, cortical interneuron density was affected mainly by the maternal *Mthfr* genotype in a region-specific manner.

**Table 2:** Interneuron densities in the hippocampus.

GAD65	Experiment 1			Univariate analysis		
Region	Layer	wt:wt:wt (*n* = 5)	ht:wt:wt (*n* = 6)	ht:wt:ht. (*n* = 5)	OG effect	MG effect
CA1	SO	0.361 ± 0.022	0.348 ± 0.072	0.506 ± 0.066	*F*_1,15_ = 4.86, *p* = 0.046	NS
CA1	SP	1.077 ± 0.105	1.683 ± 0.295	1.288 ± 0.13	*F*_1,15_ = 4.27, *p* = 0.059	*F*_1,15_ = 10.07, *p* = 0.007
CA1	SR	0.291 ± 0.050	0.298 ± 0.051	0.350 ± 0.039	NS	NS
CA1	All	0.442 ± 0.044	0.517 ± 0.084	0.551 ± 0.013	NS	NS
CA3	SO	0.372 ± 0.101	0.427 ± 0.119	0.273 ± 0.085	NS	NS
CA3	SP	0.688 ± 0.158	0.620 ± 0.149	0.897 ± 0.137	NS	NS
CA3	SR	0.687 ± 0.133	0.584 ± 0.102	0.476 ± 0.065	NS	NS
CA3	All	0.567 ± 0.066	0.524 ± 0.093	0.539 ± 0.074	NS	NS
DG	SM	0.163 ± 0.065	0.107 ± 0.025	0.097 ± 0.010	NS	NS
DG	SG	0.416 ± 0.026	0.543 ± 0.145	0.792 ± 0.083	NS	NS
DG	Hillus	0.908 ± 0.170	0.892 ± 0.202	0.666 ± 0.224	NS	NS
DG	All	0.328 ± 0.046	0.325 ± 0.056	0.349 ± 0.035	*F*_1,15_ = 3.3, *p* = 0.092	NS

PV	Experiment 1			Univariate analysis		
Region	Layer	wt:wt:wt (*n* = 4)	ht:wt:wt (*n* = 5)	ht:wt:ht. (*n* = 5)	OG effect	MG effect
CA1	SO	0.191 ± 0.049	0.123 ± 0.023	0.169 ± 0.036	NS	
CA1	SP	0.49 ± 0.134	0.689 ± 0.079	0.718 ± 0.143	NS	
CA1	SR	0.033 ± 0.014	0.013 ± 0.013	0.014 ± 0.014	NS	
CA1	All	0.181 ± 0.045	0.208 ± 0.017	0.236 ± 0.049	NS	
CA3	SO	0.256 ± 0.087	0.144 ± 0.056	0.112 ± 0.062	NS	
CA3	SP	0.326 ± 0.127	0.308 ± 0.158	0.726 ± 0.258	NS	
CA3	SR	0.185 ± 0.078	0.063 ± 0.049	0.064 ± 0.057	NS	
CA3	All	0.271 ± 0.084	0.133 ± 0.055	0.304 ± 0.095	NS	
DG	SG	0.312 ± 0.096	0.209 ± 0.088	0.185 ± 0.091	NS	
DG	Hillus	0.866 ± 0.35	0.071 ± 0.071	0.506 ± 0.341	NS	*F*_1,13_ = 3.98, *p* = 0.071
DG	All	0.423 ± 0.129	0.162 ± 0.076	0.214 ± 0.055	NS	*F*_1,13_ = 6.11, *p* = 0.031

SST	Experiment 1			Univariate analysis		
Region	Layer	wt:wt:wt (*n* = 4)	ht:wt:wt (*n* = 4)	ht:wt:ht. (*n* = 4)	OG effect	MG effect
CA1	SO	0.114 ± 0.032	0.1 ± 0.025	0.112 ± 0.029	NS	NS
CA1	SP	0.065 ± 0.025	0.021 ± 0.019	0.084 ± 0.035	NS	NS
CA1	SR	0.009 ± 0.009	0.016 ± 0.007	0.003 ± 0.003	NS	NS
CA1	All	0.052 ± 0.01	0.051 ± 0.015	0.052 ± 0.014	NS	NS
CA3	SO	0.107 ± 0.06	0.043 ± 0.02	0.031 ± 0.017	NS	NS
CA3	SP	0.21 ± 0.079	0.06 ± 0.02	0.085 ± 0.042	NS	*F*_1,10_ = 4.48, *p* = 0.067
CA3	SR	0.008 ± 0.008	0.038 ± 0.01	0.03 ± 0.023	NS	NS
CA3	All	0.105 ± 0.039	0.048 ± 0.014	0.046 ± 0.021	NS	NS
DG	SM	0.005 ± 0.003	0.006 ± 0.006	0.017 ± 0.007	NS	NS
DG	SG	0.044 ± 0.019	0.022 ± 0.009	0.023 ± 0.013	NS	NS
DG	Hillus	0.122 ± 0.051	0.151 ± 0.057	0.163 ± 0.075	NS	NS
DG	All	0.031 ± 0.009	0.027 ± 0.011	0.03 ± 0.011	NS	NS

The densities of interneurons (cells/micrometer^2^*1,000) immuno-stained for GAD65-tdTomato, PV and SST in hippocampal sub-regions are shown for experimental groups 1–3. Statistical values for the effects of offspring and maternal genotypes on interneuron densities are noted where significant. Hippocampal images were divided by field and cellular region as follows: CA1 and CA3 images were divided into the stratum oriens (SO), stratum pyramidale (SP) and stratum radiatum (SR) layers, and dentate gyrus (DG) images were divided into the hillus, stratum granulare (SC) and stratum moleculare (SM) layers; offspring *Mthfr* genotype (OG); maternal *Mthfr* genotype (MG); Not significant (NS).

### Effects of *Mthfr+/−* genotype on PV and SST interneurons

**Figure 4:**
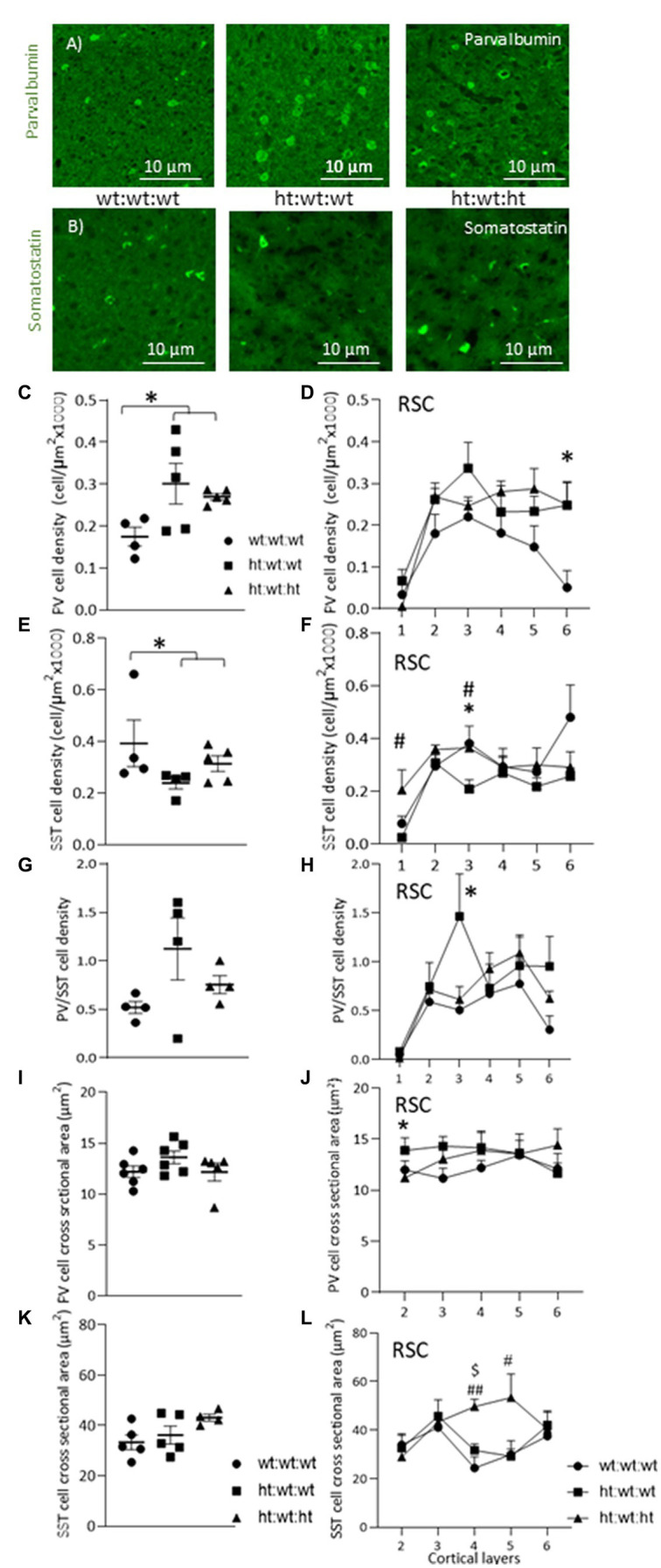
Effects of *Mthfr+/−* genotype on cortical PV and SST interneurons. **(A,B)** An example of PV and SST interneurons in Layer 3 of the RSC in the different experimental groups. **(C,D)** PV+ cell density was increased by maternal *Mthfr+/−* genotype (*F*_1,13_ = 7.19, *p* = 0.021, two-way ANOVA). **(D)** The alteration in PV+ cell density was seen throughout the entire cortical depth, with the most prominent change seen in L6 (*F*_1,11_ = 5.32, *p* = 0.042, ANOVA for repeated measures, *F*_1,13_ = 6.8, *p* = 0.024, two-way ANOVA, respectively). **(E)** SST+ cell density in the RSC. **(F)** When dividing the cortex into layers, offspring *Mthfr+/−* genotype increased and maternal *Mthfr+/−* genotype decreased SST+ cell density in layer 3 (*F*_1,12_ = 7.09, *p* = 0.024 and *F*_1,12_ = 7.79, *p* = 0.019, two-way ANOVA, respectively). **(G)** *Cortical PV/STT ratio in the RSC*. **(H)** Separate analyses of each layer showed that maternal *Mthfr+/−* genotype increased the PV/SST ratio in layer 3 (*F*_1,10_ = 6.14, *p* = 0.038, two-way ANOVA). **(I)** Average PV+ cell body cross sectional area **(J)** PV+ cell body cross sectional area was increased by the maternal *Mthfr+/−* genotype in layer 2 of the RSC (*F*_1,16_ = 4.82, *p* = 0.045, two-way ANOVA). **(K)** Average SST+ cell body cross sectional area. **(L)** SST+ cell body cross sectional area was increased by offspring *Mthfr+/−* genotype in layers 4 and 5 of the RSC (*F*_1,13_ = 11.66, *p* = 0.006 and *F*_1,13_ = 6.96, *p* = 0.023, two-way ANOVA respectively). Data are presented as means ± SEM. *N* = 6 groups 1, 2, and 3, respectively. One-way ANOVA with a Bonferroni *post-hoc* test *p < 0.05 between wt:wt:wt and ht.:wt:wt. #*p* < 0.05 between wt:wt:wt and ht:wt:ht., ##*p* < 0.01 between wt:wt:wt and ht.:wt:ht., $*p* < 0.05 between ht.:wt:wt and ht.:wt:ht.

Two major populations of interneurons in the cortex, PV and SST, are associated with a variety of external and internal input processing tasks that are performed by the cerebral cortex. Examples of PV and SST interneuron immunostaining in the RSC of all experimental groups are shown in Figures 4A,B. The maternal *Mthfr+/−* genotype was associated with an increase in total PV interneuron density (*F*_1,13_ = 7.19, *p* = 0.021, Figure 4C) that was also observed throughout the layers of the RSC (*F*_1,11_ = 5.32, *p* = 0.042, ANOVA for repeated measures), where a stronger effect was found in layer 6 (*F*_1,13_ = 6.8, *p* = 0.024, Figure 4D). In the FC, in contrast, these effects were not observed (Supplementary Figures S4A-D). In the DG region of the hippocampus, the maternal *Mthfr+/−* genotype was associated with a greater than 50% decrease in the density of PV (*F*_1,13_ = 6.11, *p* = 0.031, see Table 2).

Total SST interneuron density under the maternal *Mthfr+/−* genotype tended overall to decrease by maternal *Mthfr+/−* genotype (*F*_1,12_ = 3.84, *p* = 0.079), but contrasting layer specific effects were observed: the offspring *Mthfr+/−* genotype increased SST densities in layers 1 and 3 (*F*_1,12_ = 5.76, *p* = 0.037 and *F*_1,12_ = 7.09, *p* = 0.024, respectively), and the maternal *Mthfr+/−* genotype decreased them in cortical layer 3 compared to the offspring of *Mthfr+/+* dams (*F*_1,12_ = 7.79, *p* = 0.019, Figures 4E,F). Consistent with this finding, SST interneuron density in the FC was reduced by the maternal *Mthfr+/−* genotype relative to the *Mthfr+/+* genotype in layer 3 (*F*_1,12_ = 7.45, *p* = 0.021, Supplementary Figures S4E,F). The analysis of SST interneuron densities in the hippocampus is presented in Table 2.

The PV/SST ratio represents the functional balance between the two major interneuron populations in the cerebral cortex, each of which is active in a different functional compartment of the glutamatergic projection neurons. A higher PV/SST ratio in layer 3 was associated with the maternal *Mthfr+/−* genotype (*F*_1,10_ = 6.14, *p* = 0.038, see Figures 4G,H).

Cellular volume is affected by physiological and pathophysiological processes. To estimate the effects of the *Mthfr* genotype on interneuron volumes, we measured the cross-sectional areas of PV and SST interneurons in the RSC, the brain regions in which our analyses revealed the strongest effects on interneuron densities. The maternal *Mthfr+/−* genotype was associated with increased soma cross sectional area in PV interneurons in layer 2 (*F*_1,16_ = 4.82, *p* = 0.045) compared to *Mthfr+/+* offspring (Figures 4I,J). The offspring *Mthfr+/−* genotype was associated with increased soma cross sectional area in SST interneurons in deeper layers, layers 4 and 5, of the RSC (*F*_1,13_ = 11.66, *p* = 0.006 and *F*_1,13_ = 6.96, *p* = 0.023, respectively), suggesting that the SST interneurons in these layers swelled, see Figures 4K,L.

Similar analyses in the *Mthfr+/+* and *Mthfr+/−* offspring of *Mthfr+/+* dams did not expose any effect of offspring genotype on interneuron sub-populations in the RSC, FC and limited effect on PV density in the CA1 region of the hippocampus (Supplementary Figure S5; Supplementary Table S3). Thus, the effect of offspring genotype could be detected when interacting with deficient maternal *in utero* environment.

### ASD-like phenotype and cortical interneurons

**Figure 5:**
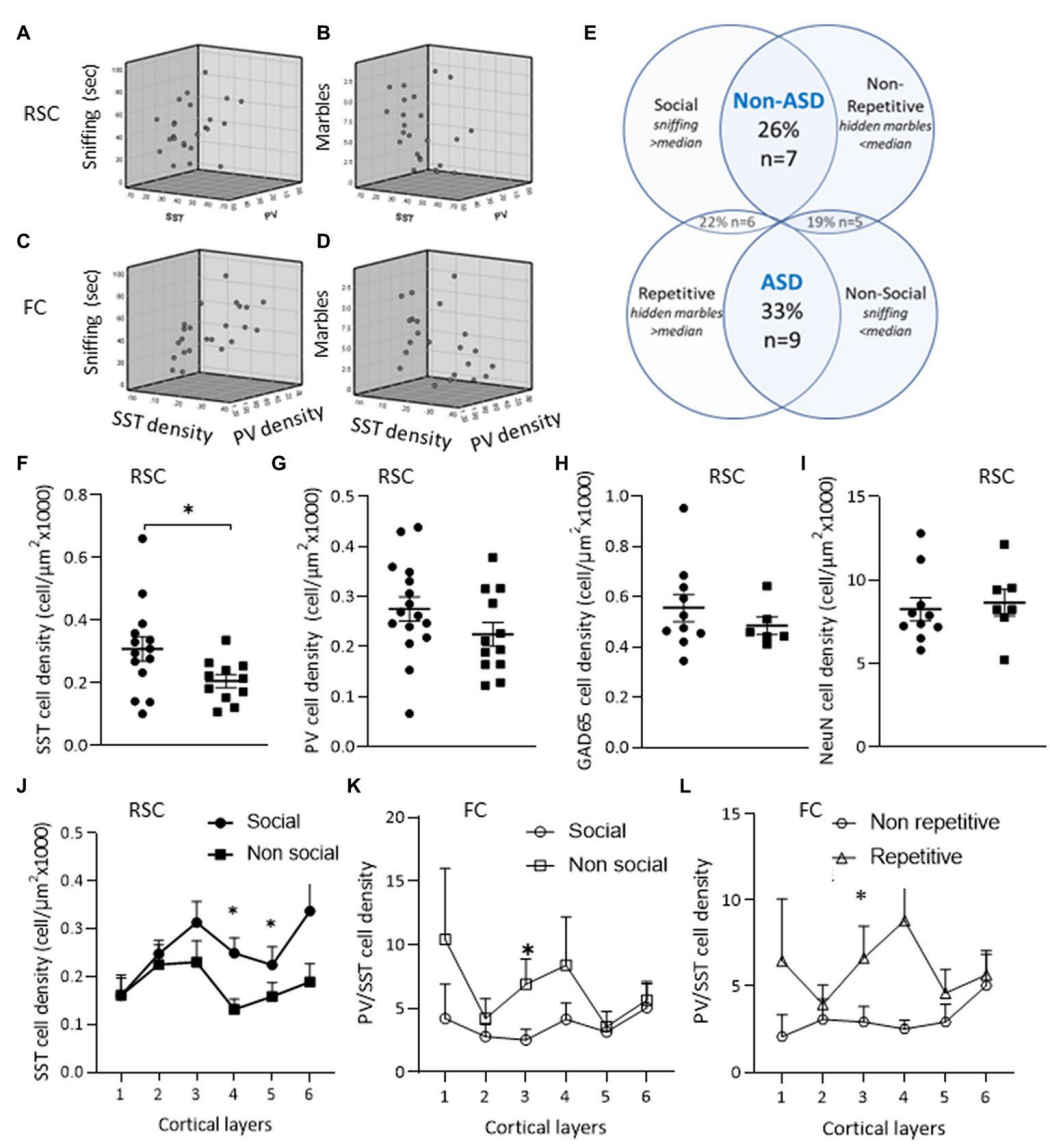
ASD-like phenotype and cortical interneurons. Relations between sniffing duration and number of hidden marbles with SST and PV interneuron densities in the RSC **(A,B)** and in the FC **(C,D)**. **(E)** Mice phenotypes – mice were grouped by behavior as described in the methods. **(F–J)** Neuron densities in the RSC of the “Non-social” compared to the “Social” group: **(F)** SST+ cell density. **(G)** PV+ cell density. **(H)** Cortical cell densities of GAD65-tdTomato interneurons. **(I)** Density of the entire neuron population. **(J)** When analyzing each cortical layer separately, the decrease in SST+ cell density was observed in layers 4–6 of the RSC of the “Non-social” compared to the “Social” group **(K,L)** PV+/SST+ ratio in FC. **(K)** An increase in PV/SST ratio in layer 3 of the “Non-social” compared to the “Social” group. **(L)** An increase in PV+/SST+ ratio in layer 3 of the “Repetitive” compared to the “Non-repetitive” group. Data are presented as means ± SEM, *N* = 13 per group. *p < 0.05 student t-test between “Social” and “Non-social” groups, between “Non-ASD-like” and “ASD-like” and between “Non-repetitive” and “Repetitive” groups.

Evaluation of the correlation between the observed phenotype and interneuron appearance found positive correlations (Table 3) between total SST interneuron density in the RSC and sniffing behavior (*R* = 0.502, *p* < 0.01) and total neuron density in the RSC and repetitive behavior (*R* = 0.528, *p* < 0.04). The correlation between SST interneuron density and behavior was more prominent in the FC, where we found a positive correlation with sniffing and a negative correlation with repetitive behavior (*R* = 0.666, *p* = 0.001 and *R* = −0.456, *p* = 0.03, respectively, Figures 5A–D, see also Supplementary Figure S6). We also evaluated whether the PV/SST ratio correlates with behavioral phenotype. A stronger correlation was observed in the FC, the PV/SST ratio negatively correlated with sniffing (*R* = −0.7) and positively correlated with repetitive behavior (*R* = 0.4, see Table 3).

**Table 3:** Correlation between cortical GABAergic interneuron density and mice behavior.

Area	Behavior		PV	SST	PV/STT	GAD	NeuN	GAD/NeuN
RSC	Sniffing	Correlation coefficient	0.205	0.502	−0.246	0.336	−0.034	0.272
		Sig. (2-tailed)	0.305	**0.011**	0.247	0.204	0.904	0.327
		N	27	25	24	16	15	15
	Hidden marbles	Correlation coefficient	−0.036	−0.364	0.264	0.135	0.528	0.024
		Sig. (2-tailed)	0.858	0.074	0.212	0.619	**0.043**	0.933
		N	27	25	24	16	15	15
FC			PV	SST	PV/SST	GAD	NeuN	GAD/NeuN
	Sniffing	Correlation coefficient	−0.120	0.666	−0.715	0.058		
		Sig. (2-tailed)	0.551	**0.001**	**0.000**	0.851	NA	NA
		N	27	22	22	13		
	Hidden marbles	Correlation coefficient	−0.064	−0.456	0.431	−0.136		
		Sig. (2-tailed)	0.753	**0.033**	**0.045**	0.659	NA	NA
		N	27	22	22	13		

Spearman’s correlation between core autistic features and cortical cell density was tested. PV, SST, GAD65 cells and the entire neuron population (NeuN) were tested. Not available (NA). Bold font indicates statistical significance.

To assess whether a particular behavioral phenotype was enriched, all of the experimental mice were categorized by their social and repetitive behaviors. Thirty three percent of the mice presented an ASD-like phenotype, exhibiting both impaired social behavior and increased repetitive behavior, while about 40% of the mice presented only one of these behavioral features (see Figure 5E). The correlation of SST interneuron appearance in the RSC with social behavior was also obtained by the lower SST densities observed in non-social compared to social mice, while PV, GAD65-tdTomato, and NeuN neuron densities did not differ in social vs. non-social mice (Figures 5F–J).

To assess whether interneuron densities in a particular layer of the RSC and FC regions contributed to the observed behaviors, the correlation was calculated by cortical layer (Table 4). SST densities in layers 3 and below and layers 2 and below in the RSC and FC regions, respectively, positively correlated with sniffing behavior. Moreover, the negative correlation of PV/SST ratio with sniffing was observed in most cortical layers, while the positive correlation with repetitive behavior was limited mainly to the contribution of FC layer 3 (Table 4 and Figures 5K,L).

**Table 4:** Correlation between cortical interneuron laminar density and ASD-like behavior.

Area	SST			L1	L2	L3	L4	L5	L6
RSC	Spearman’s rho	Sniffing	Correlation coefficient	−0.081	0.227	0.431	0.532	0.476	0.468
			Sig. (2-tailed)	0.700	0.275	**0.032**	**0.006**	**0.016**	**0.018**
			N	25	25	25	25	25	25
		Hidden marbles	Correlation coefficient	−0.197	−0.294	−0.297	−0.327	−0.240	−0.250
			Sig. (2-tailed)	0.346	0.154	0.150	0.111	0.249	0.228
			N	25	25	25	25	25	25
	PV/SST			L1	L2	L3	L4	L5	L6
	Spearman’s rho	Sniffing	Correlation coefficient	0.123	−0.210	−0.238	0.011	0.035	−0.203
			Sig. (2-tailed)	0.566	0.325	0.262	0.958	0.872	0.342
			N	24	24	24	24	24	24
		Hidden marbles	Correlation coefficient	0.126	0.295	0.223	0.125	0.121	0.032
			Sig. (2-tailed)	0.556	0.162	0.296	0.562	0.574	0.881
			N	24	24	24	24	24	24
FC	SST			L1	L2	L3	L4	L5	L6
	Spearman’s rho	Sniffing	Correlation coefficient	0.264	0.545	0.604	0.592	0.566	0.599
			Sig. (2-tailed)	0.236	**0.009**	**0.003**	**0.004**	**0.006**	**0.003**
			N	22	22	22	22	22	22
		Hidden marbles	Correlation coefficient	−0.262	−0.528	−0.480	−0.517	−0.367	−0.460
			Sig. (2-tailed)	0.239	**0.012**	**0.024**	**0.014**	**0.093**	**0.031**
			N	22	22	22	22	22	22
	PV/SST			L1	L2	L3	L4	L5	L6
	Spearman’s rho	Sniffing	Correlation coefficient	−0.577	−0.532	−0.760	−0.497	−0.283	−0.620
			Sig. (2-tailed)	**0.005**	**0.011**	**0.000**	**0.019**	**0.202**	**0.002**
			N	22	22	22	22	22	22
		Hidden marbles	Correlation coefficient	0.196	0.318	0.451	0.396	0.360	0.303
			Sig. (2-tailed)	0.383	0.149	**0.035**	0.068	0.100	0.170
			N	22	22	22	22	22	22

Spearman’s correlation between core autistic features and interneuron density was tested in each layer of the RSC and FC. Layer (L). Bold font indicates statistical significance.

The use of correlation analysis to evaluate the link between behavioral phenotype and interneuron distribution highlighted the contribution to the endophenotype made by FC interneuron organization.

### Hints of the synaptic contribution to behavioral phenotype

**Figure 6:**
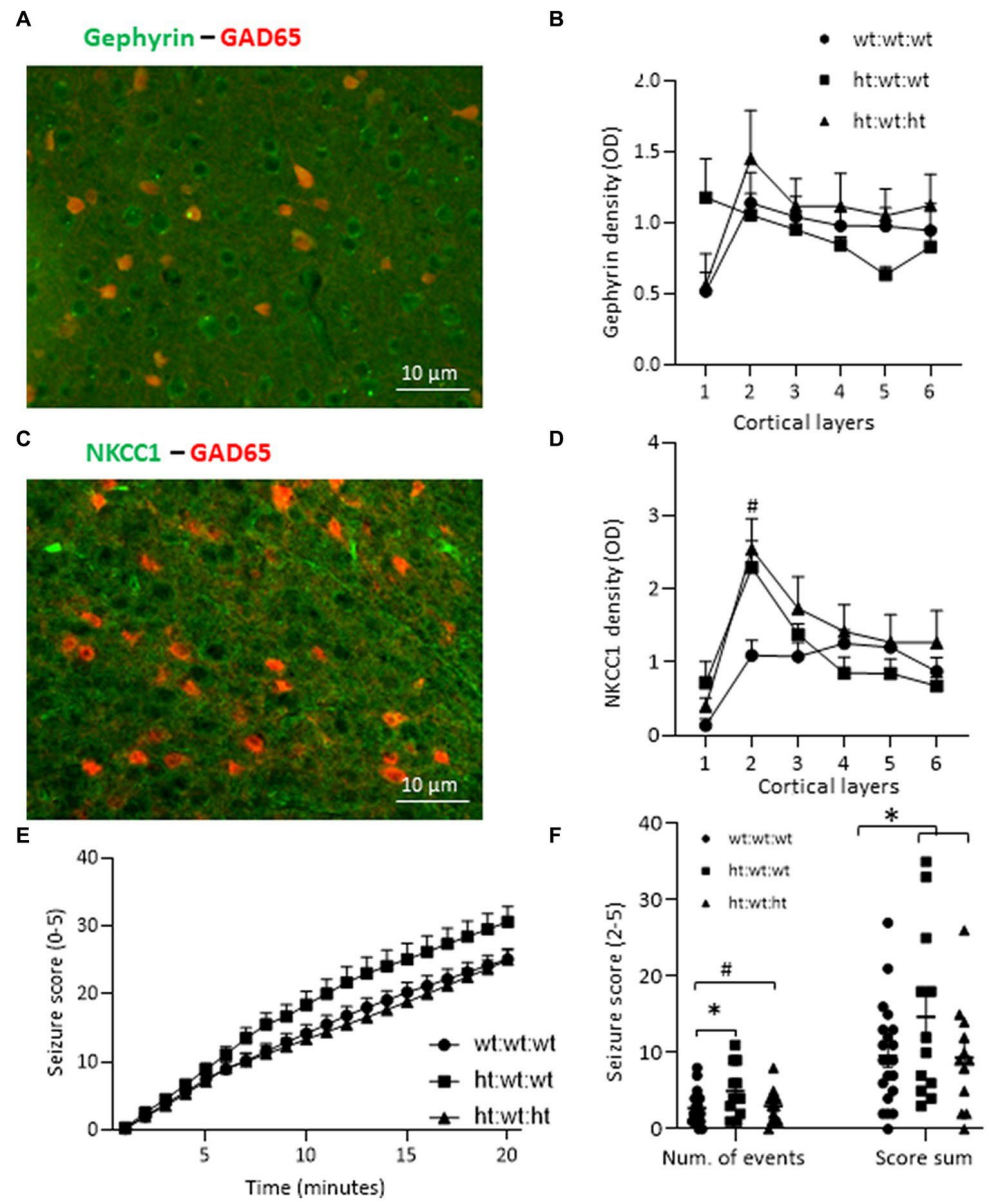
Effects of Mthfr genotype on mice susceptibility to seizure and molecular markers. **(A)** Example of gephyrin and GAD65-tdTomato in layer 3 of the RSC. **(B)** *Gephyrin density* in the RSC cortex. **(C)** Example of *NKCC1* and GAD65-tdTomato in Layer 3 of the RSC. **(D)** *NKCC1 density* was increased by the maternal *Mthfr+/−* genotype in layer 2 of the RSC (*F*_1,16_ = 5.45, *p* = 0.035, two-way ANOVA). **(E,F)** Recordings of seizure scores for each minute after PTZ injection. **(E)** *Cumulative seizure score* for each minute of the 20-min test was enhanced by the maternal *Mthfr+/−* genotype and reduced by the offspring *Mthfr+/−* genotype (*F*_1,55_ = 6.06, *p* = 0.017, *F*_1,55_ = 8.99, *p* = 0.004, respectively, ANOVA for repeated measurements). *N* = 6 per group. One-way ANOVA with a Bonferroni *post-hoc* test #*p* < 0.05 between wt:wt:wt and ht.:wt:het. **(F)** *Number of seizure events*. The number of minutes during which the mouse’s number of seizure events was in the range of 2–5. The number of seizure events was increased by maternal *Mthfr+/−* genotype and decreased by offspring *Mthfr+/−* genotype (*F*_1,58_ = 5.47, *p* = 0.023, *F*_1,58_ = 4.96, *p* = 0.03, respectively, two-way ANOVA). *The sum of the score for seizure events* was also increased by maternal genotype (*F*_1,58_ = 4.71, *p* = 0.034, two-way ANOVA). Data are presented as means ± SEM. One way ANOVA with Bonferroni *post-hoc* test *p < 0.05 between wt:wt:wt and ht.:wt:wt. N: wt:wt:wt = 21, ht.:wt:wt = 12, ht.:wt:ht. = 12.

The inhibitory tone in general depends on interneuron density, distribution and synaptic connectivity. Evaluation of the immunoreactivity of gephyrin as a marker for GABAergic postsynaptic clusters found similar levels of the protein in the RSC of all groups (Figures 6A,B). We then examined the levels of a sodium-potassium-chloride transporter, NKCC1, which helps control the intracellular chloride equilibrium. The contribution of NKCC1 to normal neurological function is exemplified by the positive effect of its antagonist, bumetanide, in neurological conditions (Löscher and Kaila, 2022). Layer specific analysis revealed that the maternal *Mthfr+/−* genotype was associated with enhanced NKCC1 levels in layer 2 of the RSC (Figures 6C,D), while NKCC1 levels in layer 1 negatively correlated with repetitive behavior (*R* = −0.67, Table 5). Evaluation by behavioral phenotype exposed a negative correlation between gephyrin levels in RSC layers 5 and 6 and social behavior (*R* = −0.69 and *R* = −0.54, respectively, Table 5), associating inhibitory synapses that populate the deep cortical layers with social phenotype.

**Table 5:** Cortical markers of inhibitory synapse, laminar density by phenotype.

Protein	Behavior	Cortical layers	L1	L2	L3	L4	L5	L6
NKCC1	Social	Correlation coefficient	−0.150	−0.186	−0.475	−0.386	−0.425	−0.468
		Sig. (2-tailed)	0.595	0.508	0.074	0.156	0.114	0.079
		N	15	15	15	15	15	15
	Hidden marbles	Correlation coefficient	−0.674	−0.280	0.136	0.097	0.368	0.150
		Sig. (2-tailed)	**0.012**	0.355	0.658	0.753	0.215	0.626
		N	13	13	13	13	13	13
Gephyrin			L1	L2	L3	L4	L5	L6
	Social	Correlation coefficient	0.100	0.007	−0.418	−0.471	−0.692	−0.539
		Sig. (2-tailed)	0.723	0.980	0.121	0.076	**0.006**	**0.038**
		N	15	15	15	15	14	15
	Hidden marbles	Correlation coefficient	0.055	0.141	0.271	0.114	0.463	0.083
		Sig. (2-tailed)	0.857	0.645	0.370	0.712	0.111	0.787
		N	13	13	13	13	13	13

Spearman’s correlation between the chloride transporter NKCC1, the GABA receptors anchoring protein, gephyrin density, and core autistic features, was tested in each layer of the RSC and FC. Layer (L). Bold font indicates statistical significance.

### Effects of *Mthfr* genotype on mice susceptibility to seizure

Interneuron defects are a major cause of epilepsy, a neurological disorder with high comorbidity in ASD (Bozzi et al., 2018). We evaluated whether an *Mthfr* deficiency altered the susceptibility of mice to a PTZ induced seizure. The cumulative seizure score for each minute of the 20-min test is presented in Figure 6C. Higher seizure scores were induced by the maternal *Mthfr+/−* genotype and lower scores were induced by the offspring *Mthfr+/−* genotype (*F*_1,55_ = 6.06, *p* = 0.017, *F*_1,55_ = 8.99, *p* = 0.004, respectively, ANOVA for repeated measurements). Seizure recurrence (number of seizure events, score 2–5) was increased by the maternal *Mthfr+/−* genotype and decreased by the offspring *Mthfr+/−* genotype (*F*_1,58_ = 5.47, *p* = 0.023, *F*_1,58_ = 4.96, *p* = 0.03, respectively). The sum of the score for seizure events was also increased by the maternal *Mthfr+/−* genotype (*F*_1,58_ = 4.71, *p* = 0.034, Figure 6D). Similar analyses in *Mthfr+/+* and *Mthfr+/−* offspring to *Mthfr+/+* dams did not show any effect of offspring genotype on the susceptibility to PTZ induced seizure (Supplementary Figure S7).

## Discussion

Maternal deficiency in MTHFR, a key regulatory enzyme of the folate cycle, caused a robust interneuron deficiency in the RSC that was reflected to lesser extents in two of its input sources. The maternal *Mthfr+/−* genotype was the main factor that affected interneuron quantity and laminar distribution, i.e., the total interneuron population and its largest sub-types, PV and SST. Furthermore, major domains of ASD-like behavior significantly correlated with SST interneuron distributions and PV/SST ratios in the RSC and FC. The functional relevance of interneuron defects to brain excitability was shown by an elevated response to the convulsant agent, PTZ.

Attempts to identify the genetic origin of a neurodevelopmental disorder typically focus on the genotype of the affected subject. Interactions between a genetic factor and the environment are more difficult to dissect. The maternal *Mthfr* genetic deficiency constitutes an example of a gene–environment interaction that results in modification of the *in-utero* environment and subsequent nutrient availability for the developing fetus by an impaired gene. The separate contributions of the offspring and maternal genotypes and that of the interaction between the two profoundly affect developmental trajectories in the brain and result in a variable spectrum of phenotypes. In humans, the maternal homozygous *Mthfr677TT* polymorphism was shown to increase the risk for ASD in the child (James et al., 2006; Goin-Kochel et al., 2009; Mohammad et al., 2009; Liu et al., 2011; Schmidt et al., 2011; Guo et al., 2012; Pu et al., 2013; Rai, 2016) and to interact with the child’s genotype (i.e., its ability to metabolize FA). However, the differential impact of maternal genotype on the risk for the *Mthfr677CC* newborn cannot be explored. In the current study, the *Mthfr+/−* genotype alone in the offspring of WT mothers (as tested in experiment 2) was not sufficient to modulate the tested behaviors in offspring, and it had only a minor effect on cortical interneuron lamination and susceptibility to PTZ induced seizure. As one may expect, not all brain circuits are affected similarly by maternal *Mthfr+/−* and its interaction with offspring genotype. Similar interaction was observed in the susceptibility to seizure, whisker trimming and nesting behavior of offspring, among which the WT offspring exhibited the strongest effect, suggesting the presence of compensatory mechanisms in the *Mthfr+/−* offspring. Supporting data for the notion of cell- or circuit-specific interactions between offspring and maternal genotype comprising PV quantity and the layer 3 PV/SST ratio were also collected. Considering the regulatory nature of the MTHFR enzyme, with multiple regulatory sites by other enzymes or products of the biochemical pathway, i.e., 5- methyl-tetrahydrofolate, S-adenosyl methionine and S-adenosylhomocysteine (Froese et al., 2018), it is possible that the response to the *in-utero* deficiency in the *Mthfr+/−* offspring differs from that of its WT littermate.

The reduced RSC interneuron densities observed in the offspring of *Mthfr+/−* mothers cannot be attributed solely to changes in either the PV or SST interneuron population, which undergoes more restricted change. The lower number of interneurons may be compensated for by an excessive inhibitory innervation, a possibility that was partially excluded by the restricted effects observed for gephyrin and by NKCC1 cortical density.

PV and SST interneurons differently contribute to the processing of sensory, cognitive and emotional functions (Pfeffer et al., 2013). Exerting differential and concerted regulation on cortical beta and gamma oscillations (Kato et al., 2015; Kuki et al., 2015; Chen et al., 2017; Montgomery et al., 2022), they are inversely active in response to novel vs. familiar visual stimuli (Hayden et al., 2021). The observed changes in interneuron densities and proportions in the offspring of *Mthfr+/−* dams may therefore engender modifications in mice abilities to process audio-visual stimuli during social encounters that lead to social deficits. Providing support for this notion, PV or SST ablation was sufficient to cause social and cognitive impairment, with a stronger effect obtained by SST ablation (Perez et al., 2019). Moreover, an altered PV/SST ratio was associated with poor responses to social stimuli in the Pten deficient mice model (Vogt et al., 2015; Shin et al., 2021). In addition to the quantitative changes, SST interneuron swelling in deep cortical layers may represent energetic failure (Brisson and Andrew, 2012) and/or a volumetric response to changes in cortical fluid ionic strengths that potentially involve changes in their activity (Lauderdale et al., 2015).

Although the *Mthfr* deficiency is known to confer increased risks for ASD in humans and for ASD-like features in mice, due to biological variability and *in-utero* factors, human and mice present a variable phenotype. Work to find subgroups of patients that share behavioral profiles has been done in both human research (Vargason et al., 2019; Nordahl et al., 2022) and in animal models of psychiatric disorders (El-Kordi et al., 2013; Ardi et al., 2016; Zilkha et al., 2017; Orenbuch et al., 2019), with the goal of enabling biological origin to be determined. Employing this approach, we show that SST densities in the RSC and FC positively correlated with prosocial behavior and SST density in the FC negatively correlated with repetitive behavior. An observed negative correlation between the PV/SST ratio and prosocial behavior was specific to the FC. As a whole, our analyses of brain-behavior relations enhanced our ability to detect neural correlates to behavior and to point specifically to the SST laminar organization in the RSC and FC as targets with significant translational relevance that warrants further research.

Input from the FC, hippocampus and other brain regions is integrated by the RSC and is forwarded by feedback projections to primary sensory cortices (Vann et al., 2009; Zhang et al., 2014). fMRI studies have implicated the RSC in a wide range of cognitive functions, including episodic and autobiographical memory, imagining future events, and executive function, some of which are altered in ASDs (Chan et al., 2009; Spreng et al., 2009; Vann et al., 2009; Demetriou et al., 2018). The RSC was also suggested to translate between egocentric (self-centered) and allocentric (world-centered) spatial reference frames (Marchette et al., 2014; Miller et al., 2014). Here we show that the maternal *Mthfr+/−* genotype affects both the RSC and its major input sources with a main effect on SST interneurons. The resultant lower numbers of SST interneurons in the RSC may have significant implications for network processing, social perception and behavior that can result in asocial behavior as reported here. Circuit-wise, the dendrites of principal neurons in layer 1 of the RSC integrate inputs from local inhibitory SST synapses (Tremblay et al., 2016; Urban-Ciecko and Barth, 2016) and other brain regions. Lower SST interneuron counts may result in weaker inhibitory input to layer 1, thereby interfering with processing. Although no evidence exists of such changes in the RSC of ASD patients, mice models carrying human mutations were reported to possess irregular RSC function that manifested in impaired social and sensory-motor function and that involved abnormal SST cell quantities (Vesuna et al., 2020; Yang et al., 2021). Our findings support the notion that the RSC regulates social behavior by interfering with the integration of sensorimotor information. Longer recognized for its association with ASD is the FC (Carper and Courchesne, 2005; Geschwind and Levitt, 2007; Teffer and Semendeferi, 2012; van Rooij et al., 2018), with several reports of hypoactivation during social cognition tasks (Hadjikhani et al., 2007; Yang et al., 2017). Although initially, the correlation found between sociability and SST interneurons in the FC seems to contradict this observation, considering the significant cortical interneuron innervation by the SST (Pfeffer et al., 2013), reduced SST cell quantities may result in disinhibition of PV and VIP cells, which would increase the inhibitory input to principal cells. This may also explain the lower GABA levels observed in the FC of autistic patients (Harada et al., 2011).

Insofar as interneuron quantities and distributions may affect inhibitory innervation, our initial estimations of postsynaptic elements and their cortical distributions showed layer specific effects of maternal folate metabolism and the relation of the observed distribution patterns to behavioral phenotypes. Considering these proteins as representative of inhibitory synapses, we conclude that in the tested brain regions, the effect of the *Mthfr* genotype on inhibitory synapses is restricted. Lastly, whether female offspring exhibit similar associations between social behavior and SST interneurons remains to be determined. An improved understanding of these complex interactions will require further study to elucidate interneuron connectivity.

## Data availability statement

The original contributions presented in the study are included in the article/Supplementary material, further inquiries can be directed to the corresponding author.

## Ethics statement

The animal study was reviewed and approved by the Animal Care and Use Committee of Ben-Gurion University of the Negev, Beer-Sheva, Israel.

## Author contributions

NS conceptualized, planned and performed the experiments, developed method for analysis, and analyzed the data. GS performed the experiments and data analysis. JH developed and provided the Transgene GAD65-tdTomato mice and commented on the manuscript. HG conceptualized and planned the experiments, supervised the experiments and analysis, and funded the study. NS and HG wrote the first draft of the manuscript. All authors read and approved the final manuscript.

## Funding

This study was supported by the Israel Science Foundation grant 515/17 to HG.

## Conflict of interest

The authors declare that the research was conducted in the absence of any commercial or financial relationships that could be construed as a potential conflict of interest.

## Correction note

This article has been corrected with minor changes. These changes do not impact the scientific content of the article.

## Publisher’s note

All claims expressed in this article are solely those of the authors and do not necessarily represent those of their affiliated organizations, or those of the publisher, the editors and the reviewers. Any product that may be evaluated in this article, or claim that may be made by its manufacturer, is not guaranteed or endorsed by the publisher.

## Abbreviations

ASD: autism spectrum disorder
DSI: direct social interaction
FA: folic acid
FC: frontal cortex
GABA: gamma-aminobutyric acid
GAD65: glutamate-decarboxylase 65 isoform
HT: *Mthfr+/−* heterozygote
MTHFR: methylene-tetrahydrofolate reductase protein
NeuN: neuronal enolase
NKCC1: Na^+^-K^+^-2Cl co-transporter
PV: parvalbumin
PTZ: pentylenetetrazol
RSC: retrosplenial cortex
SST: somatostatin
WT: wild-type
