# Supplementary material for: Genetic impairment of folate metabolism regulates cortical interneurons and social behavior
Source: Front Neurosci. 2023 Jun 28;17:1203262. doi: 10.3389/fnins.2023.1203262 (PMC10338116; doi:10.3389/fnins.2023.1203262)
Supplement: Supplementary file 1 [file Presentation_1.pdf]

Supplementary Material

Genetic Impairment of Folate Metabolism Regulates Cortical Interneurons and Social Behavior

Noa Sadigurschi[1], Gilad Scrift[1], Johannes Hirrlinger[2], Hava M. Golan[1,3]

1. Department of Physiology and Cell Biology, Faculty of Health Sciences, Ben-Gurion University of the Negev
2. Carl-Ludwig-Institute for Physiology, University of Leipzig, Germany
3. Azrieli National Center for Autism and Neurodevelopment Research, Ben-Gurion University of the Negev

Corresponding author:

Hava M. Golan

Mail: havag@bgu.ac.il

Supplementary Figure 1.

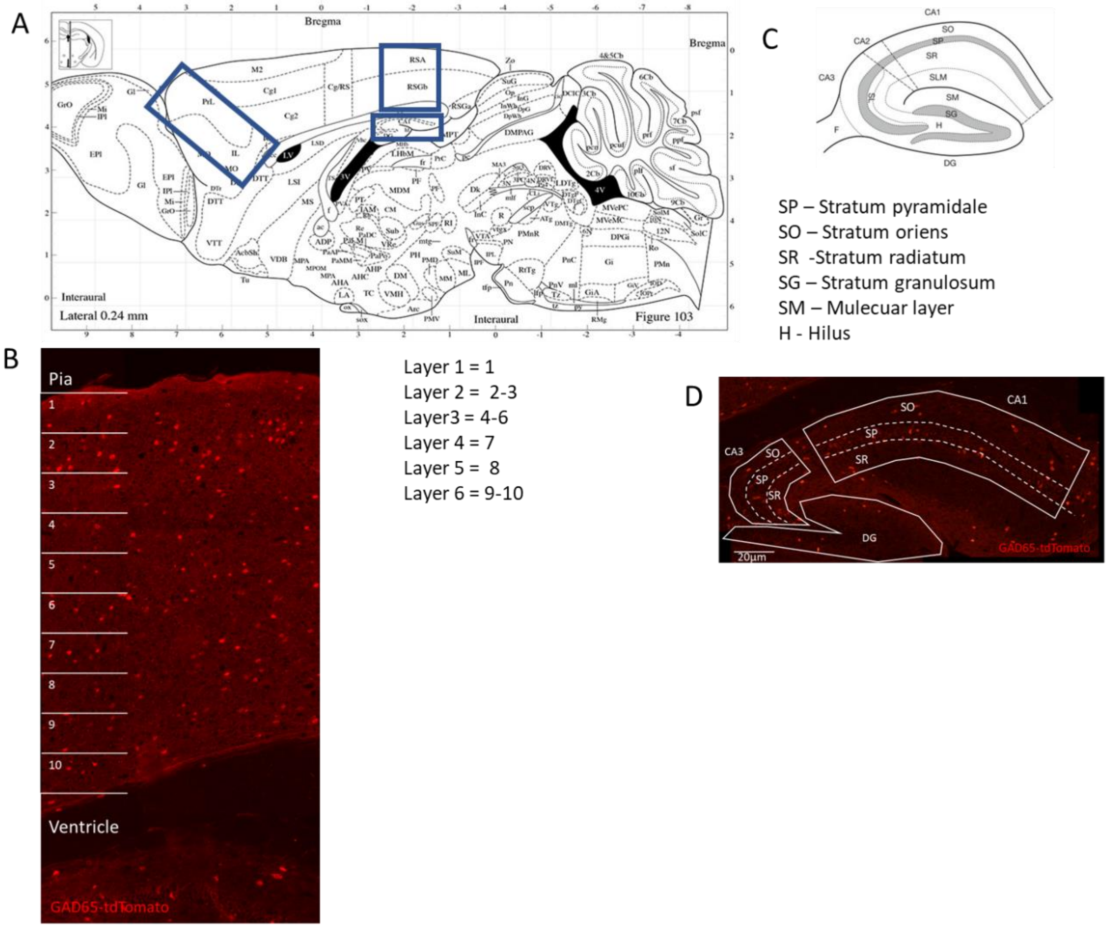

Supplementary Figure 1 - Immunofluorescence analysis. Images of the frontal cortex (FC), retrosplenial cortex (RSC) and hippocampus regions were captured from each brain section. (A) Brain regions were identified according to the protocol of Paxinos and Franklin (Mouse Brain in Stereotaxic Coordinates, 2004). (B) For the analysis of the FC and RSC, cortical images from the pia to the ventricle were combined and divided into 10 equally sized bins for cell density and fluorescence optical density analyses. (C and D) Hippocampal images were divided by fields and cellular region as follows: CA1 and CA3 were divided into the stratum oriens (SO), stratum pyramidale (SP) and stratum radiatum (SR), and the dentate gyrus (DG) was divided into the hillus and stratum granulare (SC) and stratum moleculare (SM).

Supplementary Figure 2.

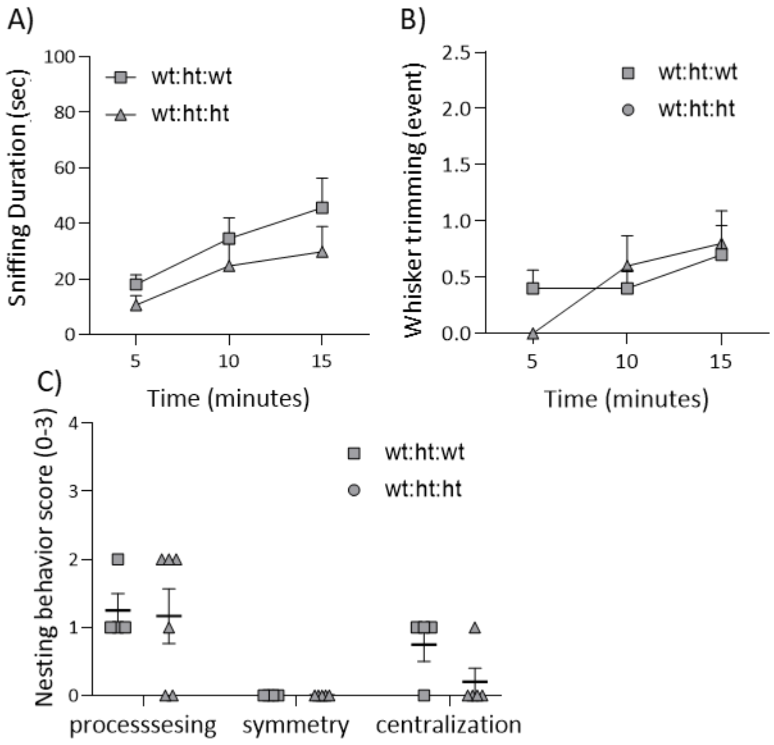

Supplementary Figure 2: Effect of offspring genotype on adult mouse behavior.

Mthfr+/+ and Mthfr+/- offspring of Mthfr+/+ dams presented similar behavior. A. Sniffing duration in the direct social interactions (DSI) test. B. Whiskers trimming during DSI test, and C. Nest material processing.

Supplementary Figure 3.

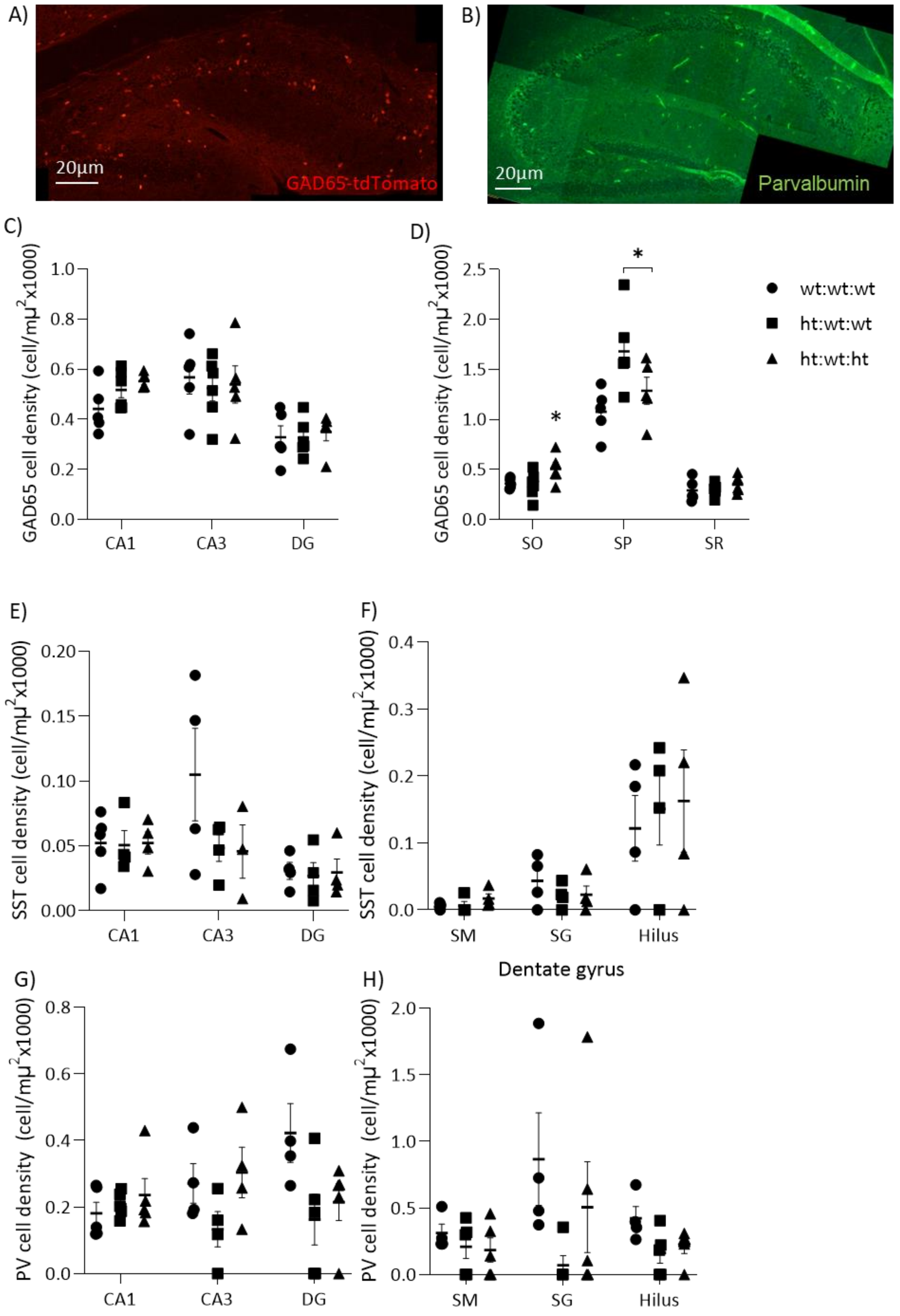

Supplementary Figure 3 – *Effect of Mthfr+/- genotype on GABAergic cell density in the hippocampus.*

*A-B) Images of GAD65-tdTomato neurons and of PV interneurons, respectively, in the hippocampus. C) GAD65-tdTomato cell density in the hippocampus. D) When analyzing CA1 sub-regions separately, the offspring Mthfr+/- genotype was found to increase GAD65-tdTomato cell density in the SO layer of the CA1 region and the maternal Mthfr+/- genotype increased cell density in the SP layer of CA1 ($F_{1,15}$=4.86 p=0.046, $F_{1,15}$=10.07 p=0.007, two-way ANOVA, respectively). SST interneurons density in the hippocampus (E) and in the different layers of the DG (F). G-H) PV interneuron density was deceased in the DG by the maternal Mthfr+/- genotype ($F_{1,13}$=6.11 p=0.031, two-way ANOVA, respectively) (G). PV interneurons density in DG layers (F). Data are presented as means ± SEM, N of group 1=5, of group 2=5, of group 3=5, One-way ANOVA with a Bonferroni post-hoc test *= $p < 0.05$. Cornu ammonis (CA); Dentate gyrus (DG); Stratum pyramidale (SP); Stratum oriens (SO); Stratum radiatum (SR) Stratum granulosum (SG); Stratum moleculare layer (SM); Hilus (H); Parvalbumin (PV); Somatostatin (SST).*

Supplementary Figure 4.

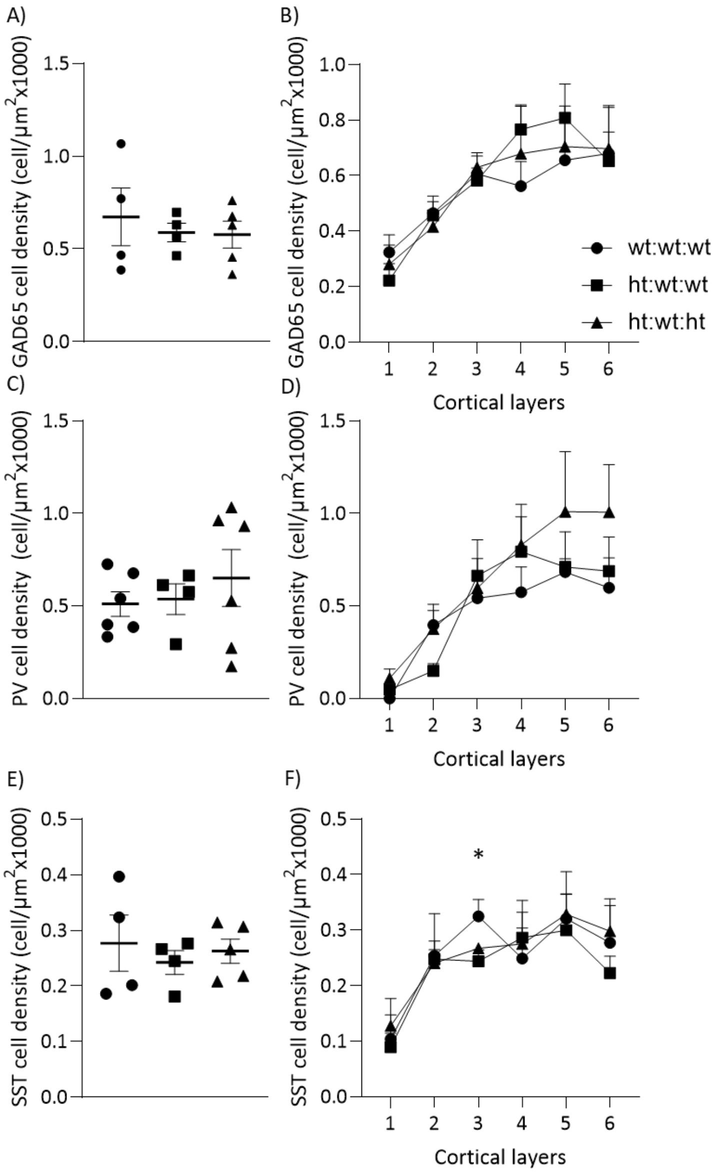

Supplementary Figure 4 – Effect of Mthfr+/- genotype on GAD65, PV and SST interneuron densities in the frontal cortex.

A-B) *GAD interneuron density* over the entire depth of the FC (A) and in each layer individually (B), **C-D)** *PV interneuron density* over the entire depth of the FC (C) and in each layer individually (D), **E-F)** *SST interneuron density* over the entire depth of the frontal cortex in experiment 1 (E). Analysis of each layer individually showed that SST interneuron densities were decreased by the maternal Mthfr+/- genotype in layer 3 of the frontal cortex ($F_{1,12}$=7.45, p=0.021, two-way ANOVA) (F). Data are presented as means ± SEM**.** N of group 1=5, group 2=5, of group 3=6. Parvalbumin (PV); Somatostatin (SST).

Supplementary Figure 5.

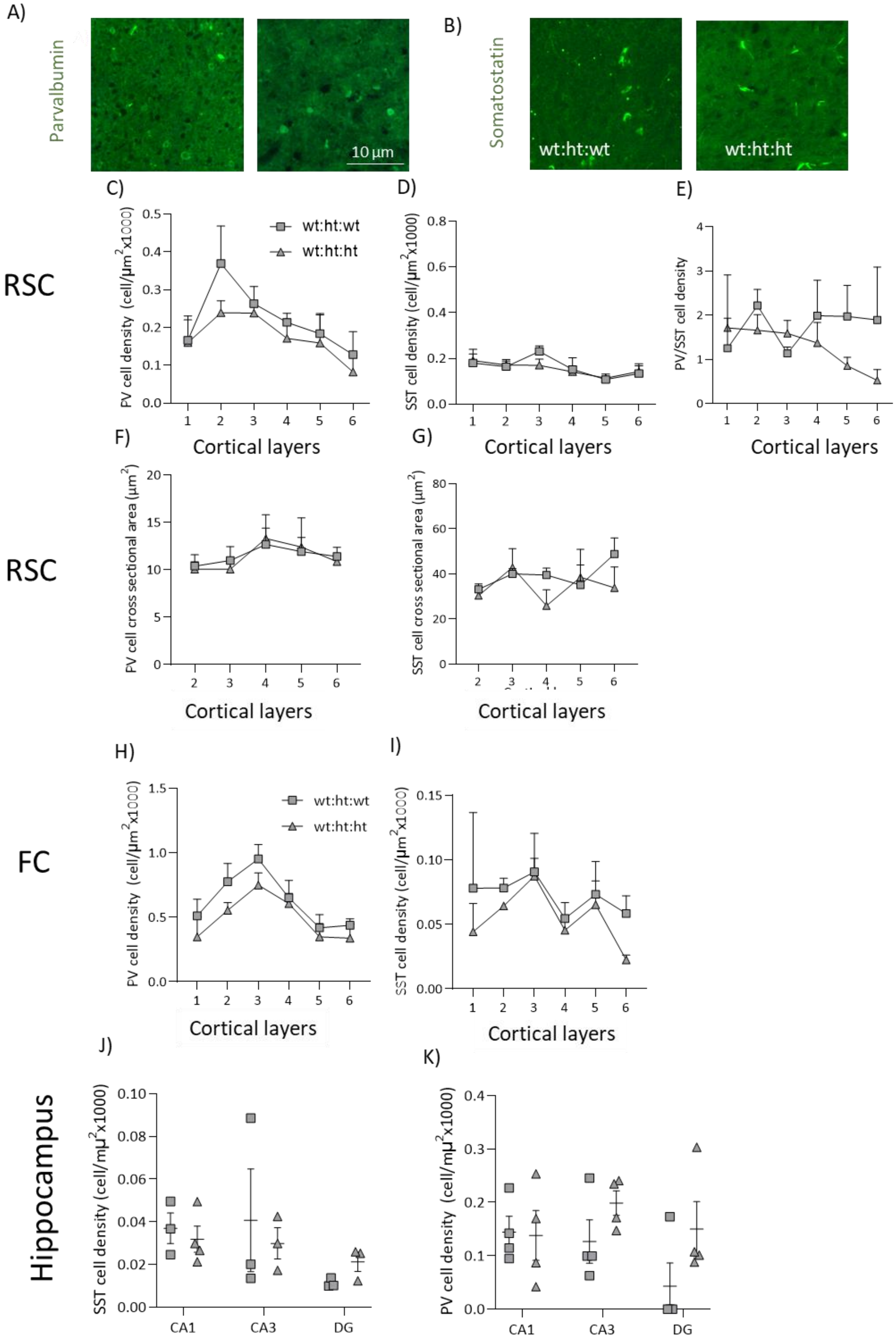

Supplementary Figure 5 **–** Effect of Mthfr+/- genotype on interneuron density in the offspring of Wt mother (Experiment 2).

A, B) Images of PV and SST immunostaining in layer 3 of the RSC of offspring of Mthfr+/+dams and Mthfr+/- males. Interneuron laminar density; **C)** PV, **D)** SST, **E)** PV/SST and interneuron cross sectional area; **F)** PV and **G)** SST. PV and SST interneurons density in the Frontal cortex **(H-I)**, and in the hippocampus **(J- K)**. Data are presented as means ± SEM. N of group 4=4; group 5=5. Parvalbumin (PV); Somatostatin (SST).

Supplementary Figure 6

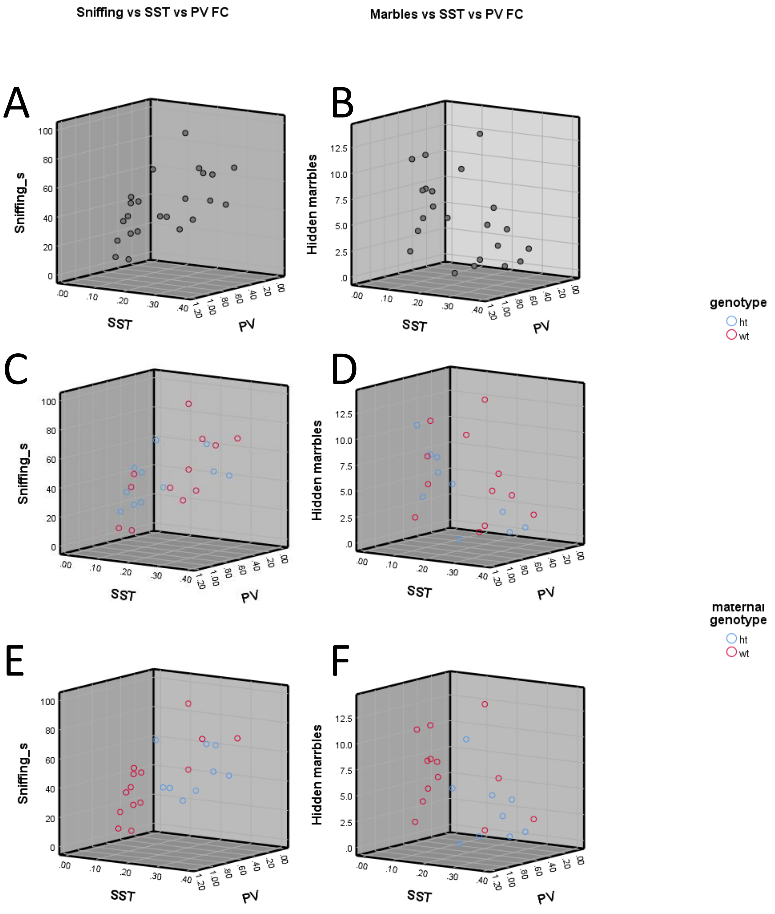

***Supplementary Figure 6** – ASD-like phenotype and cortical interneurons*.

Relations between sniffing duration and number of hidden marbles with SST and PV interneuron densities in the FC (A and B) are presented. Data presented for the offspring *Mthfr+/-* genotype (C and D) and for the maternal *Mthfr+/-* genotype (E and F). Mthfr+/- = Blue, WT = red.

Supplementary Figure 7.

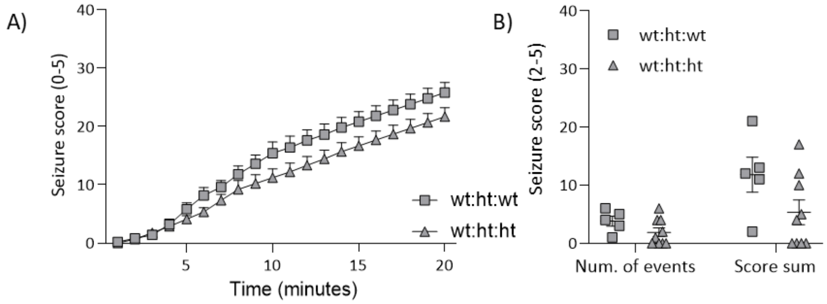

Supplementary Figure 7 – Effect of offspring Mthfr genotype on its response to PTZ in the offspring of Wt mother.

Recordings of seizure scores for each minute after PTZ injection. (A) The cumulative seizure score for each minute of the 20-minute test. (B) Number of seizure events. The number of minutes in which the mouse scored in the range of 2-5 seizure events. Sum of the seizure score. N= Data are presented as means ± SEM.

Supplementary Table 1

Supplementary Table 1 - Direct social interaction, at 5, 10 and 15 min of the test.

Experiment	Experiment 1										Experiment 2					
MG		wt	wt	wt	het	het	het	het	het	het	wt	wt	wt	wt	wt	wt
PG		wt	wt	wt	wt	wt	wt	wt	wt	wt	het	het	het	het	het	het
OG		wt	wt	wt	wt	wt	wt	het	het	het	wt	wt	wt	het	het	het
Group		1	1	1	2	2	2	3	3	3	4	4	4	5	5	5
**Stat**	**Variable**	**N**	**AVG**	**SD**	**N**	**AVG**	**SD**	**N**	**AVG**	**SD**	**N**	**AVG**	**SD**	**N**	**AVG**	**SD**
NN	5Count	6	0.667	0.816	8	1.000	1.069	8	1.375	1.302	10	1.000	0.943	10	0.500	0.707
	5Dur	6	1.667	2.251	8	1.500	1.927	8	1.500	1.309	10	1.000	0.943	10	0.600	0.966
	10Count	6	1.000	1.549	8	0.500	0.926	8	0.500	0.756	10	0.600	0.699	10	0.700	0.949
	10Dur	6	3.333	6.282	8	0.625	1.188	8	0.625	1.061	10	0.800	0.919	10	1.600	2.271
	15Count	6	0.833	1.169	8	0.500	0.535	8	0.500	0.756	10	0.500	0.527	10	0.200	0.422
	15Dur	6	2.333	2.733	8	0.500	0.535	8	0.500	0.756	10	0.600	0.699	10	0.200	0.422
NH	5Count	6	0.500	0.548	8	0.250	0.707	8	1.000	0.926	10	2.400	1.578	10	1.100	1.287
	5Dur	6	1.333	1.506	8	0.500	1.414	8	1.625	1.506	10	4.900	5.763	10	1.300	1.567
	10Count	6	1.000	1.265	8	1.625	1.188	8	1.875	1.458	10	1.600	1.430	10	1.100	1.287
	10Dur	6	3.333	4.131	8	4.750	4.132	8	7.375	5.069	10	4.300	4.620	10	2.400	3.836
	15Count	6	1.667	1.633	8	0.750	0.886	8	0.875	0.991	10	2.000	2.000	10	0.900	1.101
	15Dur	6	10.667	11.656	8	6.250	8.328	8	2.500	4.000	10	7.700	12.212	10	3.200	4.050
NA	5Count	6	2.833	2.563	8	2.125	1.808	8	2.125	1.356	10	3.000	2.055	10	1.200	1.229
	5Dur	6	6.500	9.182	8	4.000	4.504	8	3.625	2.973	10	4.900	4.332	10	2.200	2.440
	10Count	6	1.833	2.639	8	1.375	1.061	8	1.875	0.641	10	1.600	1.506	10	0.900	1.449
	10Dur	6	8.667	13.706	8	2.375	2.134	8	6.625	4.809	10	2.900	3.213	10	2.100	3.929
	15Count	6	2.167	2.639	8	0.500	0.926	8	2.250	1.982	10	1.200	1.687	10	1.100	1.197
	15Dur	6	5.333	7.685	8	1.250	2.375	8	5.625	6.093	10	2.800	4.264	10	1.700	1.889
BS	5Count	6	2.000	1.897	8	2.500	1.414	8	3.500	2.330	10	3.000	2.309	10	2.400	2.757
	5Dur	6	6.167	5.419	8	3.875	2.475	8	7.625	6.632	10	7.200	7.162	10	6.500	9.846
	10Count	6	3.000	2.828	8	2.500	1.309	8	2.500	1.690	10	3.200	3.155	10	2.900	2.424
	10Dur	6	13.333	15.253	8	7.000	5.345	8	5.250	3.694	10	8.500	14.486	10	8.000	10.088
	15Count	6	2.333	2.251	8	2.500	2.070	8	2.375	2.066	10	2.000	1.633	10	2.400	2.366
	15Dur	6	10.500	11.113	8	17.000	32.262	8	11.000	13.867	10	5.300	5.832	10	7.200	10.809
Sniffing	5Count	6	6.000	4.336	8	5.875	3.603	8	8.000	3.891	10	9.400	3.718	10	5.200	3.967
	5Dur	6	15.667	12.941	8	9.875	7.568	8	14.375	8.766	10	18.000	11.005	10	10.600	10.564
	10Count	6	6.833	3.656	8	6.000	2.330	8	6.750	3.284	10	7.000	3.916	10	5.600	4.033
	10Dur	6	28.667	17.154	8	14.750	6.840	8	19.875	10.467	10	16.500	17.501	10	14.100	14.851
	15Count	6	7.000	3.899	8	4.250	2.712	8	6.000	4.243	10	5.700	4.191	10	4.600	3.893
	15Dur	6	18.333	11.308	8	8.000	8.036	8	8.625	7.230	10	11.100	14.067	10	5.100	5.705
CU	5Count	6	0.000	0.000	8	0.625	1.061	8	0.375	0.744	10	0.600	0.966	10	0.700	0.949
	10Count	6	0.333	0.516	8	0.000	0.000	8	0.500	0.926	10	0.300	0.483	10	0.300	0.483
	15Count	6	0.500	0.837	8	0.000	0.000	8	0.000	0.000	10	0.200	0.422	10	0.100	0.316
CO	5Count	6	0.167	0.408	8	0.375	0.518	8	0.125	0.354	10	0.300	0.483	10	0.500	1.269
	10Count	6	0.500	0.837	8	0.000	0.000	8	0.500	0.926	10	0.400	0.699	10	0.300	0.483
	15Count	6	0.333	0.516	8	0.125	0.354	8	0.125	0.354	10	0.100	0.316	10	0.100	0.316
WT	5Count	6	0.000	0.000	8	0.000	0.000	8	0.000	0.000	10	0.400	0.516	10	0.000	0.000
	10Count	6	0.167	0.408	8	0.875	0.835	8	0.000	0.000	10	0.000	0.000	10	0.600	0.843
	15Count	6	0.167	0.408	8	0.875	1.126	8	0.125	0.354	10	0.300	0.675	10	0.200	0.422
	Dur5	6	0.000	0.000	8	0.000	0.000	8	0.000	0.000	10	0.000	0.000	10	0.000	0.000
	Dur10	6	3.667	5.680	8	0.000	0.000	8	0.000	0.000	10	0.000	0.000	10	0.100	0.316
	Dur15	6	3.667	5.680	8	0.000	0.000	8	11.000	20.368	10	0.000	0.000	10	0.100	0.316
Non Aggre	5Count	6	6.167	4.401	8	6.875	3.271	8	8.500	3.854	10	10.700	3.974	10	6.400	4.904
	5Dur	6	15.833	12.983	8	10.875	7.080	8	14.875	8.493	10	19.300	11.156	10	11.800	10.891
	10Count	6	8.167	4.355	8	6.875	2.031	8	7.750	4.268	10	7.700	4.347	10	6.900	5.109
	10Dur	6	33.333	21.833	8	15.625	6.823	8	20.875	10.092	10	17.200	17.542	10	15.400	14.864
	15Count	6	8.000	4.195	8	5.250	3.454	8	6.750	4.166	10	6.300	4.739	10	5.000	4.190
	15Dur	6	19.333	12.127	8	9.000	8.281	8	19.875	20.863	10	11.700	14.545	10	5.500	5.968
A	5Count	6	0.000	0.000	8	0.625	1.188	8	0.125	0.354	10	0.000	0.000	10	0.300	0.675
	5Dur	6	0.000	0.000	8	1.250	2.550	8	0.375	1.061	10	0.000	0.000	10	0.900	2.025
	10Count	6	0.000	0.000	8	0.500	1.069	8	0.125	0.354	10	0.300	0.949	10	1.000	1.333
	10Dur	6	0.000	0.000	8	1.250	2.375	8	0.625	1.768	10	1.200	3.795	10	2.500	3.536
	15Count	6	0.167	0.408	8	0.250	0.707	8	0.250	0.707	10	0.000	0.000	10	0.600	1.075
	15Dur	6	0.167	0.408	8	0.625	1.768	8	0.625	1.768	10	0.000	0.000	10	2.800	5.329
F	5Count	6	0.000	0.000	8	0.125	0.354	8	0.000	0.000	10	0.000	0.000	10	0.200	0.422
	5Dur	6	0.000	0.000	8	0.250	0.707	8	0.000	0.000	10	0.000	0.000	10	0.500	1.269
	10Count	6	0.000	0.000	8	0.000	0.000	8	0.000	0.000	10	0.000	0.000	10	0.000	0.000
	10Dur	6	0.000	0.000	8	0.000	0.000	8	0.000	0.000	10	0.000	0.000	10	0.000	0.000
	15Count	6	0.667	1.033	8	0.000	0.000	8	0.000	0.000	10	0.000	0.000	10	0.000	0.000
	15Dur	6	2.667	4.131	8	0.000	0.000	8	0.000	0.000	10	0.000	0.000	10	0.000	0.000

Direct social behavior between two mice of the same group. Each behavior was quantified for its duration (Dur) and the number of events (Count) at 5, 10 and 15 min of the test.
The following behaviors were analyzed: NN = Nose tip-to-nose tip; NH = Nose-to-head; NA = Nose-to-anogenital; BS = Body sniffing; Sniffing = sum of all sniffing behaviors; CO = Crawl Over; CU = Crawl Under; WT = Whisker trimming;
OG = Offspring Mthfr genotype; MG = maternal Mthfr genotype; PG = maternal Mthfr genotype.

Supplementary Table 2

Supplementary Table 2 - Direct social interaction, Cumulative number and duration of events.																
Experiment		Experiment 1									Experiment 2					
MG		wt	wt	wt	het	het	het	het	het	het	wt	wt	wt	wt	wt	wt
PG		wt	wt	wt	wt	wt	wt	wt	wt	wt	het	het	het	het	het	het
OG		wt	wt	wt	wt	wt	wt	het	het	het	wt	wt	wt	het	het	het
Group		1	1	1	2	2	2	3	3	3	4	4	4	5	5	5
Stat	Variable	N	AVG	SD	N	AVG	SD	N	AVG	SD	N	AVG	SD	N	AVG	SD
NN Comul	Count5	6	0.667	0.816	8	1.000	1.069	8	1.375	1.302	10	1.000	0.943	10	0.500	0.707
	Count 10	6	1.667	1.862	8	1.500	1.604	8	1.875	1.246	10	1.600	0.843	10	1.200	1.229
	Count15	6	2.500	2.811	8	2.000	1.927	8	2.375	1.188	10	2.100	0.994	10	1.400	1.174
	Dur5	6	1.667	2.251	8	1.500	1.927	8	1.500	1.309	10	1.000	0.943	10	0.600	0.966
	Dur10	6	5.000	6.450	8	2.125	2.416	8	2.125	1.356	10	1.800	1.033	10	2.200	2.974
	Dur15	6	7.333	8.017	8	2.625	2.774	8	2.625	1.302	10	2.400	1.430	10	2.400	2.875
NH Comul	Count5	6	0.500	0.548	8	0.250	0.707	8	1.000	0.926	10	2.400	1.578	10	1.100	1.287
	Count 10	6	1.500	1.378	8	1.875	1.642	8	2.875	1.885	10	4.000	2.211	10	2.200	1.989
	Count15	6	3.167	2.858	8	2.625	2.264	8	3.750	2.605	10	6.000	3.944	10	3.100	2.644
	Dur5	6	1.333	1.506	8	0.500	1.414	8	1.625	1.506	10	4.900	5.763	10	1.300	1.567
	Dur10	6	4.667	3.670	8	5.250	5.339	8	9.000	5.210	10	9.200	8.904	10	3.700	3.917
	Dur15	6	15.333	12.691	8	11.500	12.513	8	11.500	7.597	10	16.900	19.365	10	6.900	6.557
NA Comul	Count5	6	2.833	2.563	8	2.125	1.808	8	2.125	1.356	10	3.000	2.055	10	1.200	1.229
	Count 10	6	4.667	3.386	8	3.500	2.507	8	4.000	1.512	10	4.600	3.134	10	2.100	2.025
	Count15	6	6.833	5.981	8	4.000	2.449	8	6.250	2.550	10	5.800	3.853	10	3.200	2.860
	Dur5	6	6.500	9.182	8	4.000	4.504	8	3.625	2.973	10	4.900	4.332	10	2.200	2.440
	Dur10	6	15.167	14.261	8	6.375	5.927	8	10.250	5.776	10	7.800	5.789	10	4.300	4.572
	Dur15	6	20.500	21.824	8	7.625	6.022	8	15.875	8.560	10	10.600	7.457	10	6.000	5.793
BS Comul	Count5	6	2.000	1.897	8	2.500	1.414	8	3.500	2.330	10	3.000	2.309	10	2.400	2.757
	Count 10	6	5.000	4.648	8	5.000	2.507	8	6.000	3.381	10	6.200	4.984	10	5.300	3.945
	Count15	6	7.333	5.538	8	7.500	3.703	8	8.375	4.596	10	8.200	5.160	10	7.700	5.964
	Dur5	6	6.167	5.419	8	3.875	2.475	8	7.625	6.632	10	7.200	7.162	10	6.500	9.846
	Dur10	6	19.500	20.394	8	10.875	5.743	8	12.875	8.576	10	15.700	20.276	10	14.500	19.004
	Dur15	6	30.000	27.151	8	27.875	33.310	8	23.875	20.301	10	21.000	21.674	10	21.700	29.470
Sniffing Co	Count5	6	6.000	4.336	8	5.875	3.603	8	8.000	3.891	10	9.400	3.718	10	5.200	3.967
	Count 10	6	12.833	6.646	8	11.875	5.643	8	14.750	6.159	10	16.400	6.501	10	10.800	7.223
	Count15	6	19.833	9.988	8	16.125	7.453	8	20.750	8.876	10	22.100	9.562	10	15.400	10.772
	Dur5	6	15.667	12.941	8	9.875	7.568	8	14.375	8.766	10	18.000	11.005	10	10.600	10.564
	Dur10	6	44.333	20.646	8	24.625	12.141	8	34.250	17.119	10	34.500	23.773	10	24.700	25.096
	Dur15	6	62.667	31.232	8	32.625	16.758	8	42.875	21.067	10	45.600	33.649	10	29.800	28.751
CU Comul	Count5	6	0.000	0.000	8	0.625	1.061	8	0.375	0.744	10	0.600	0.966	10	0.700	0.949
	Count 10	6	0.333	0.516	8	0.625	1.061	8	0.875	1.458	10	0.900	1.287	10	1.000	1.333
	Count15	6	0.833	1.329	8	0.625	1.061	8	0.875	1.458	10	1.100	1.449	10	1.100	1.449
CO Comul	Count5	6	0.167	0.408	8	0.375	0.518	8	0.125	0.354	10	0.300	0.483	10	0.500	1.269
	Count 10	6	0.667	1.211	8	0.375	0.518	8	0.625	0.916	10	0.700	0.675	10	0.800	1.549
	Count15	6	1.000	1.265	8	0.500	0.535	8	0.750	0.886	10	0.800	0.632	10	0.900	1.595
WT Comu	Count5	6	0.000	0.000	8	0.000	0.000	8	0.000	0.000	10	0.400	0.516	10	0.000	0.000
	Count 10	6	0.167	0.408	8	0.875	0.835	8	0.000	0.000	10	0.400	0.516	10	0.600	0.843
	Count15	6	0.333	0.516	8	1.750	1.753	8	0.125	0.354	10	0.700	0.823	10	0.800	0.919
	Dur5	6	0.000	0.000	8	0.000	0.000	8	0.000	0.000	10	0.000	0.000	10	0.000	0.000
	Dur10	6	3.667	5.680	8	0.000	0.000	8	0.000	0.000	10	0.000	0.000	10	0.100	0.316
	Dur15	6	3.667	5.680	8	0.000	0.000	8	11.000	20.368	10	0.000	0.000	10	0.100	0.316
Non Aggre	Count5	6	6.167	4.401	8	6.875	3.271	8	8.500	3.854	10	10.700	3.974	10	6.400	4.904
	Count 10	6	14.333	7.202	8	13.750	5.120	8	16.250	6.475	10	18.400	7.442	10	13.300	9.117
	Count15	6	22.333	11.112	8	19.000	7.231	8	23.000	8.848	10	24.700	11.136	10	18.300	13.047
	Dur5	6	15.833	12.983	8	10.875	7.080	8	14.875	8.493	10	19.300	11.156	10	11.800	10.891
	Dur10	6	49.167	24.895	8	26.500	11.892	8	35.750	16.202	10	36.500	24.001	10	27.200	25.324
	Dur15	6	68.500	36.336	8	35.500	16.518	8	55.625	32.522	10	48.200	34.637	10	32.700	29.402
A Comulat	Count5	6	0.000	0.000	8	0.625	1.188	8	0.125	0.354	10	0.000	0.000	10	0.300	0.675
	Count 10	6	0.000	0.000	8	1.125	2.100	8	0.250	0.707	10	0.300	0.949	10	1.300	1.947
	Count15	6	0.167	0.408	8	1.375	2.669	8	0.500	1.414	10	0.300	0.949	10	1.900	2.514
	Dur5	6	0.000	0.000	8	1.250	2.550	8	0.375	1.061	10	0.000	0.000	10	0.900	2.025
	Dur10	6	0.000	0.000	8	2.500	4.899	8	1.000	2.828	10	1.200	3.795	10	3.400	5.060
	Dur15	6	0.167	0.408	8	3.125	5.793	8	1.625	4.596	10	1.200	3.795	10	6.200	8.753
F Comulat	Count5	6	0.000	0.000	8	0.125	0.354	8	0.000	0.000	10	0.000	0.000	10	0.200	0.422
	Count 10	6	0.000	0.000	8	0.125	0.354	8	0.000	0.000	10	0.000	0.000	10	0.200	0.422
	Count15	6	0.667	1.033	8	0.125	0.354	8	0.000	0.000	10	0.000	0.000	10	0.200	0.422
	Dur5	6	0.000	0.000	8	0.250	0.707	8	0.000	0.000	10	0.000	0.000	10	0.500	1.269
	Dur10	6	0.000	0.000	8	0.250	0.707	8	0.000	0.000	10	0.000	0.000	10	0.500	1.269
	Dur15	6	2.667	4.131	8	0.250	0.707	8	0.000	0.000	10	0.000	0.000	10	0.500	1.269

irect social behavior between two mice of the same group. Each behavior was quantified for its duration (Dur) and the number of events (Count) at 5, 0 and 15 min of the test.
he following behaviors were analyzed: NN = Nose tip-to-nose tip; NH = Nose-to-head; NA = Nose-to-anogenital; BS = Body sniffing; Sniffing = sum of all niffing behaviors; CO = Crawl Over; CU = Crawl Under; WT = Whisker trimming;
G = Offspring Mthfr genotype; MG = maternal Mthfr genotype; PG = maternal Mthfr genotype.

Supplementary Table 3

PV				
		Experiment 2		t test
Region	Layer	wt:ht:wt (n=4)	wt:ht:ht (n=4)	
CA1	SO	0.163±0.017	0.213±0.083	NS
CA1	SP	0.294±0.035	0.256±0.093	NS
CA1	SR	0.066±0.045	0.023±0.012	NS
CA1	All	0.145±0.029	0.138±0.051	NS
CA3	SO	0.117±0.051	0.096±0.052	NS
CA3	SP	0.167±0.057	0.454±0.175	NS
CA3	SR	0.069±0.069	0.203±0.119	NS
CA3	All	0.127±0.04	0.199±0.054	NS
DG	SG	0±0	0.116±0.05	0.06
DG	Hillus	0.109±0.109	0.284±0.091	NS
DG	All	0.043±0.043	0.15±0.056	NS
**SST**				
		Experiment 2		t test
Region	Layer	wt:ht:wt (n=3)	wt:ht:ht (n=3)	
CA1	SO	0.087±0.034	0.067±0.022	NS
CA1	SP	0.023±0.013	0.014±0.005	NS
CA1	SR	0.002±0.002	0.013±0.003	0.019
CA1	All	0.037±0.012	0.032±0.009	NS
CA3	SO	0.035±0.017	0.02±0.011	NS
CA3	SP	0.083±0.059	0.01±0.008	NS
CA3	SR	0.021±0.011	0.056±0.018	0.051
CA3	All	0.041±0.023	0.03±0.011	NS
DG	SM	0.004±0.003	0.014±0.005	0.054
DG	SG	0±0	0±0	NS
DG	Hillus	0.07±0.021	0.095±0.037	NS
DG	All	0.011±0.003	0.021±0.007	0.097

Supplementary Table 3 – Effect of offspring *Mthfr* genotype on interneuron densities in the hippocampus.

The densities of interneurons (cells/micrometer^2*1000) immuno-stained for PV and SST in hippocampal sub-regions are shown for two experimental groups. Statistical values (two-tailed Student's t-test) for the effects of offspring genotypes on interneuron densities are noted where significant. Hippocampal images were divided by field and cellular region as follows: CA1 and CA3 images were divided into the stratum oriens (SO), stratum pyramidale (SP) and stratum radiatum (SR) layers, and dentate gyrus (DG) images were divided into the hillus, stratum granulare (SC) and stratum moleculare (SM) layers; Not significant (NS).
